# Time-series-based forecasting of accident-related referrals to Maharaj Nakorn Chiang Mai Hospital, Northern Thailand, during each year and especially the “Seven dangerous Days” periods

**DOI:** 10.1186/s12889-026-26304-9

**Published:** 2026-01-17

**Authors:** Pimwarat Srikummoon, Patrinee Traisathit, Kaweesak Chittawatanarat, Kamtone Chandacham, Areerat Kittikhunakon, Thippapha Piriyahaphan, Suttipong Kawilapat, Narain Chotirosniramit

**Affiliations:** 1 Department of Statistics, Faculty of Science, Chiang Mai University, Chiang Mai, Thailand; 2 Data Science Research Center, Department of Statistics, Faculty of Science, Chiang Mai University, Chiang Mai, Thailand; 3 Department of Surgery, Faculty of Medicine, Chiang Mai University, Chiang Mai, Thailand; 4 Medical Records and Statistics Section, Maharaj Nakorn Chiang Mai Hospital, Chiang Mai, Thailand

**Keywords:** Time series, Seven dangerous days, New year festivals, Songkran festival, Patients

## Abstract

**Background:**

Road traffic crashes are a leading cause of death and disability globally and are a significant contributor to emergency department (ED) referrals, which places a considerable burden on ED resources and staff. As a major tourist destination and the largest city in northern Thailand, Chiang Mai experiences a surge in visitors, particularly during the New Year and Songkran festivals, which are referred to as the “Seven Dangerous Days” periods. The objective of the present study was to predict the number of crash-related ED referrals at Maharaj Nakorn Chiang Mai Hospital using time-series analysis.

**Methods:**

Data from 19,164 patients referred to the ED in Maharaj Nakorn Chiang Mai Hospital between May 2007 and December 2022 were used to develop ARIMA and SARIMA models. The predicted number of referrals from these models was then compared with the observed number of referrals collected from 2023 to 2024. Furthermore, this study also examined patient referrals during the “Seven Dangerous Days” periods. The sensitivity analyses for investigation of effect of structural changes were performed by excluding the data in trained dataset before fully Trauma Referral Audit (TRA) implementation and/or after COVID-19 pandemic.

**Results:**

Motorcycle crashes were the most common cause of injury (49.8%). The SARIMA(0,1,2)(1,0,1)_12_ model provided the best fit for motorcycle and car crashes-related referrals, while the SARIMA(1,1,1)(1,0,1)_12_ model was selected for combined referrals. The results of sensitivity analysis model excluded train dataset before fully TRA complementation show the higher prediction performance than baseline model. For the “Seven Dangerous Days” periods, the ARIMA(0,1,0) and ARIMA(2,1,1) models were the most suitable for the New Year and Songkran periods, respectively.

**Conclusion:**

The time-series based patient referral forecasting model that considered for the structural changes during study period could provide useful information for several simulations of preparation for proactive hospital management. Utilizing these models after adjusting for these major events could assist hospitals and relevant agencies in high-risk areas or during festival periods by providing predictions of traffic accident-related referrals that would enable the proactive allocation of necessary staff and resources.

**Supplementary Information:**

The online version contains supplementary material available at 10.1186/s12889-026-26304-9.

## Introduction

Road traffic crashes are a leading cause of death and disability around the world [1, 2], accounting for more than 85% of all deaths and around 90% of all disabilities in developing countries [1, 3]. The danger level is particularly high for pedestrians, cyclists, and motorcyclists, who account for more than 90% of deaths from road traffic crashes in low-income countries [4, 5]. Crashes comprise a major cause of referral to emergency departments (EDs), which places a significant burden on ED resources and time [6]. The World Health Organization (WHO) has reported that road traffic crashes are a major cause of death in people under 30 years of age, with the number of road traffic deaths reaching a high of 1.19 million in 2021 [3].

Thailand has one of the highest rates of road traffic deaths in Asia, with 7,000 people dying, 15,000 people left with disabilities, and an economic loss of around US$12.5 million in 2022 [3]. Indeed, crashes are a significant public health burden in terms of both direct and indirect economic losses [7, 8]. An injury severity score (ISS) greater than 15 has been associated with mortality, disability, and lengthy hospitalization [9]. Of the 822,841 road traffic crashes occurring in 2023, most involved motorcycles (78.6%), followed by cars (8.2%), with the majority involving people aged 30–60 years old (~ 30%) [10]. In Thailand, preventive operational measures are applied during the “Seven Dangerous Days” periods (the New Year and the Thai New Year (Songkran) national holiday periods of 3–5 days) when people working in Bangkok and other major cities travel to their hometowns [11, 12]. During this time, the number of road users increases significantly, as does the number of road traffic crashes. Monitoring the latter helps to identify losses and provide information for agencies to improve overall road safety [12].

Chiang Mai (the largest city in northern Thailand) is a popular tourist destination, especially during the New Year and Songkran festivals. There were 32,192 reported road traffic crashes in the city in 2023, which is the highest number of road crashes in the northern region and is consistently among the top five areas in Thailand [10]. The average number of injuries treated in EDs between 2014 and 2019 was approximately 14,000 per year [13]. The number of road traffic crash-related injury cases treated per year in Maharaj Nakorn Chiang Mai Hospital (a leading tertiary hospital that provides both ED and inpatient services) is 200,000 [14]. At such a rate, a shortage of healthcare workers could lead to difficulty in administering services at this hospital.

Time-series models have been applied in crash prediction by many researchers [15–19]. These include autoregressive (AR) integrated moving average (MA) (ARIMA) and seasonal ARIMA (SARIMA) models, with the latter used to fit variables observed at equally spaced or nearly equally spaced time intervals [20].

The objective of the present study was to predict the number of crash-related referrals to the ED in Maharaj Nakorn Chiang Mai Hospital by using time-series analysis. This could help hospitals predict the potential number of referred trauma patients over time, thereby enabling them to allocate the necessary supplies and staff when necessary. Moreover, developing preventive measures to reduce crash-related injuries would also assist hospitals and relevant agencies in high-risk areas or during festival periods.

## Methods

### Study design and population

Data were retrospectively collected from the medical records of injured patients referred to the Emergency Department (ED) at Maharaj Nakorn Chiang Mai Hospital, Thailand, between May 2007 and December 2024. The referral protocol for transferring patients from primary or secondary healthcare facilities to Maharaj Nakorn Chiang Mai Hospital (a tertiary care center in northern Thailand) was conducted in accordance with the Trauma Referral Audit (TRA) guidelines [21]. Specifically, referral decisions were predicated on the following nine management criteria: airway management, breathing support, oxygen saturation monitoring, hemorrhage control, intravenous fluid resuscitation, electrocardiogram (EKG) monitoring, external splinting, pelvic splinting, and cervical spine protection. The evaluations were performed by on-call physicians and emergency department personnel certified in Advanced Trauma Life Support (ATLS). Consequently, the majority of referred patients presented with severe injuries, characterized by an Injury Severity Score (ISS) exceeding 9.

The injuries included those sustained by Traffic crashes, including pedestrians, cyclists, motorcycle crashes, car crashes, or from falls and other causes. In addition, the data also included information concerning sex, age, occupation, risk behavior (such as not using a seat belt or helmet, and alcohol consumption), date of admission and discharge, duration of admission, ISS, injury source, and time of referral.

### Handling of missing data

While data for the primary identifiers were complete, some of the behavioral variables, specifically helmet use, seatbelt use, and alcohol consumption, contained missing entries due to incomplete documentation. Because of the specificity of these variables, we could not impute their missing values and so conducted further analyses using only the available values. It should be noted that since these behavioral factors were not utilized as predictors in the forecasting model, their data missingness did not impact the integrity or the performance of the primary analysis.

### Time series models and forecast assessment

Time series analysis was applied to model the monthly frequency of the injured patients referred to Maharaj Nakorn Chiang Mai Hospital and to predict future incidences. SARIMA was selected due to its robustness in handling autocorrelation and seasonality commonly found in epidemiological time-series data. To this end, SARIMA is an extension of the non-seasonal ARIMA (*p*,* d*,*q*) model with incorporated seasonal components (*P*,* D*,*Q*)*s*, where *p* denotes the order of non-seasonal autoregression, *d* is the degree of non-seasonal differencing, *q* is the order of non-seasonal MA, *P* is the order of seasonal autoregression, *D* is the degree of seasonal differencing, *Q* is the order of seasonal MA, and *s* is the length of the seasonal cycle. This framework is particularly suitable for modeling seasonal and non-stationary time-series data.

Since it is important to evaluate the stationarity of a time series prior to any estimation, it was first assessed using the Augmented Dickey-Fuller (ADF) test. When non-stationarity was detected, differencing was applied to stabilize the mean and variance over time. The ARIMA (*p*,* d*,*q*) model for non-stationary time series *Y*_*t*_ can be expressed as

$$\:\phi\:\left(B\right){\left(1-{B}\right)}^{d}{Y}_{t}=\psi\:\left({B}\right){\epsilon\:}_{t},$$

where *d* is the degree of differencing, *B* is a backshift operator, $\:\phi\:\left({B}\right):1-{\phi\:}_{1}\left({B}\right)-{\phi\:}_{2}{\left({B}\right)}^{2}-\dots\:-{\phi\:}_{p}{\left({B}\right)}^{p}$ is an AR operator, $\:\psi\:\left({B}\right):1+{\psi\:}_{1}\left({B}\right)+{\psi\:}_{2}{\left({B}\right)}^{2}+\dots\:+{\psi\:}_{p}{\left({B}\right)}^{p}$ is an MA operator, *Y*_*t*_ is the number of referral patients and $\:{\epsilon\:}_{t}$ is a white-noise error term when $\:{\epsilon\:}_{t}\sim\mathrm{N}\left(0,{\sigma\:}^{2}\right)$.

Relatedly, the SARIMA (*p*,* d*,*q*)(*P*,* D*,*Q*)*s* model can be written as

$$\:{{\Phi\:}}_{P}\left({B}^{s}\right){\phi\:}_{p}\left({B}\right){\left(1-{B}\right)}^{d}{\left(1-{B}^{s}\right)}^{D}{y}_{t}={\theta\:}_{q}\left({B}\right){{\Theta\:}}_{Q}\left({B}^{s}\right){\epsilon\:}_{t},$$

where $\:{{\Phi\:}}_{P}\left({B}^{s}\right)$ and $\:{{\Theta\:}}_{Q}\left({B}^{s}\right)$ represent the seasonal AR and MA operators, respectively.

Model identification was performed by examining autocorrelation and partial autocorrelation factor plots to suggest candidate values for *p*,* d*,* q*, *P*,* D*, and *Q.* Autocorrelation Function (ACF) and Partial Autocorrelation Function (PACF) were used to identify the possible values for *p*,* d*,* q*, *P*,* D*, and *Q* after differencing transformation.

Candidate models were selected based on minimizing multiple criteria, including the root mean square error (RMSE) for predictive accuracy and the Akaike Information Criterion (AIC) and Bayesian Information Criterion (BIC) for parsimony and overall goodness-of-fit. The adequacy of the final selected model was confirmed using the Ljung-Box test to ensure that no significant autocorrelation remained in the residuals.

Forecasting performance was assessed using the mean absolute error (MAE), mean absolute percentage error (MAPE), RMSE, and prediction interval coverage at 95% (PI Cov.%). MAE is defined as$\:\:\mathrm{M}\mathrm{A}\mathrm{E}=\frac{1}{n}\sum\:_{t=1}^{n}|{y}_{t}-{\widehat{y}}_{t}|$, where $\:{y}_{t}$ is the number of referred patients and $\:{\widehat{y}}_{t}\:$is the predicted number of referred patients. MAPE is defined as

$$\:{M}{A}{P}{E}=100\times\:\frac{1}{n}\sum\limits_{t=1}^{n}\left|\frac{{y}_{t}-{\widehat{y}}_{t}}{{y}_{t}}\right|$$

and RMSE is expressed as

$$\:{R}{M}{S}{E}=\sqrt{\frac{1}{n}\sum\:_{t=1}^{n}{({y}_{t}-{\widehat{y}}_{t})}^{2}}$$

### Data analysis

Demographics of referral patients were described through frequencies and percentages for categorical variables and median and interquartile range (IQR) for continuous ones. For the time series analysis, we focused on (1) overall referral patients, (2) motorcycle crashes, and (3) car crashes. ARIMA and SARIMA models were trained on data from May 2007 – December 2022 and validated against observed data from January 2023 – December 2024 to enable the assessment of out-of-sample forecasts for a two-year period. In addition, we focused on patient referrals during the “Seven Dangerous Days” periods. The “Seven Dangerous Days” periods for the New Year and Songkran are reported in Supplementary Table S1. We used Stata 15 software for the analysis.

### Sensitivity analysis of the training windows for the total number of referrals

This was conducted to evaluate whether the robustness of the total number of referrals forecasts was affected by the COVID-19 pandemic or changes in the patient referral policy. During the study period, the main structural changes were due to the full implementation of TRA in 2011 and the COVID-19 pandemic from 2020 to 2022. Therefore, we constructed three sensitivity models as follows:

1. Model S1: Excluding data before TRA implementation, the training dataset comprised entries between January 2011 and December 2022.
2. Model S2: Excluding data after COVID-19, the training dataset comprised entries between January 2007 and December 2019.
3. Model S3: Excluding data before TRA implementation and after COVID-19, the training dataset comprised entries between January 2011 and December 2019.

We only conducted the sensitivity analysis on the total number of referrals since we assumed that referrals from both motorcycle and car crashes would yield similar results under these circumstances.

## Results

### Overall study period

As provided in Table 1, of the 19,164 patients referred to Maharaj Nakorn Chiang Mai Hospital between May 2007 and December 2022, 14,269 (74.5%) were male. The median age of the participants was 37 years (IQR: 22–54 years old). After stratifying into age groups, the highest proportion of participants was aged 20–29 years old (19.9%), 18.3% were under 20 years old, and 17.5% were aged ≥ 60 years old. The majority were laborers (42.7%), followed by students (13.3%) and the elderly (17.5%). Most referrals (34.9%) occurred between 18:00–23:59. Referrals from traffic accidents accounted for 59.9%, with motorcycle crashes being the most common cause of injury (49.8%), followed by falls (18.8%). Most referrals were within Chiang Mai (82.7%), with a median ISS of 9 (IQR: 4–22). Of the patients referred, 40.8% had consumed alcohol. Concurrently, not using a helmet and not using seat a belt at the time of the crash were 79.2% and 83.4% of cases, respectively. The overall mortality rate was 6.4%, with 93.4% occurring in the hospital and 6.7% beforehand. The mortality rates for patients referred to Maharaj Nakorn Chiang Mai Hospital who had or had not consumed alcohol were similar each year, and slightly increased in 2021 (Supplementary Fig. S1(a)). The mortality rates for patients who used or did not use a seatbelt were inconsistent: in 2021, the number of patients who used a seatbelt (12.5%) was higher than those who did not (3.4%). Moreover, there were no patient deaths for several years (Supplementary Fig. S1(b)). Interestingly, the mortality rate of patients who did not wear a helmet was higher than for those who did, especially after 2017 (Supplementary Fig. S1(c)).

**Table 1:** Demographics of patients refer to Maharaj Nakorn Chiang Mai Hospital (*N* = 19,164)

Variable	*n*(%) or median [IQR]
Sex	
Male	14,269 (74.5)
Female	4,895 (25.5)
Age (years)	37 [22–54]
< 20	3,509 (18.3)
20–29	3,805 (19.9)
30–39	2,849 (14.9)
40–49	2,693 (14.1)
50–59	2,835 (14.8)
≥ 60	3,360 (17.5)
Unknow	113 (0.6)
Occupation	
Student	2,546 (13.3)
Government officer	686 (3.6)
Employee	612 (3.2)
Agriculturist / Trade	578 (3.0)
Laborer	8,190 (42.7)
The elderly (Age ≥ 60year)	3,362 (17.5)
Unknow	3,190 (16.7)
Time of referral	
00:00–05:59	3,286 (17.2)
06:00–11:59	2,637 (13.8)
12:00–17:59	5,961 (31.1)
18:00-23.59	6,687 (34.9)
Unknow	593 (3.1)
Mechanism of injury	
Traffic accidents	11,486 (59.9)
Pedestrian	459 (2.4)
Pedal cyclist	324 (1.7)
Motorcycle crashes	9,538 (49.8)
Car crashes	1,165 (6.0)
Fall	3,600 (18.8)
Other	4,078 (21.3)
Referrals	
Within Chiang Mai	15,850 (82.7)
Outside Chiang Mai	3,314 (17.3)
ISS, median (IQR)	9 [4–22])
Used alcohol (*n* = 7,904)	3,224 (40.8)
Not wearing a helmet (*n* = 7,180)	5,688 (79.2)
Not using a seat belt (*n* = 1,372)	1,144 (83.4)
Time in hospital	
< 48 h.	1,718 (9.0)
≥ 48 h. to 7 days	5,620 (29.3)
> 7 days	8,068 (42.1)
Unknow	3,758 (19.6)
Total mortality	1,218 (6.4)
Prehospital mortality	81 (6.7)
In-hospital mortality	1,137 (93.4)

*IQR* interquartile range, *ISS* Injury Severity Score

Of the referred patients involved in crashes, the proportions of elderly and students tended to increase, while the proportion of manual laborers tended to decrease over the study period (See Supplementary Fig. S2(a)). Motorcycle-crash cases increased over time (See Supplementary Fig. 2S(c)), with the majority of them being individuals aged 20–59 years old each year (Supplementary Fig. S3). This was accompanied by fewer individuals wearing motorcycle helmets over time (See Supplementary Fig. S4(b)) and spending more than 7 days in hospital (See Supplementary Fig. S4 (d)).

### Model selection and performance prediction metrics for all periods

**Fig. 1:**
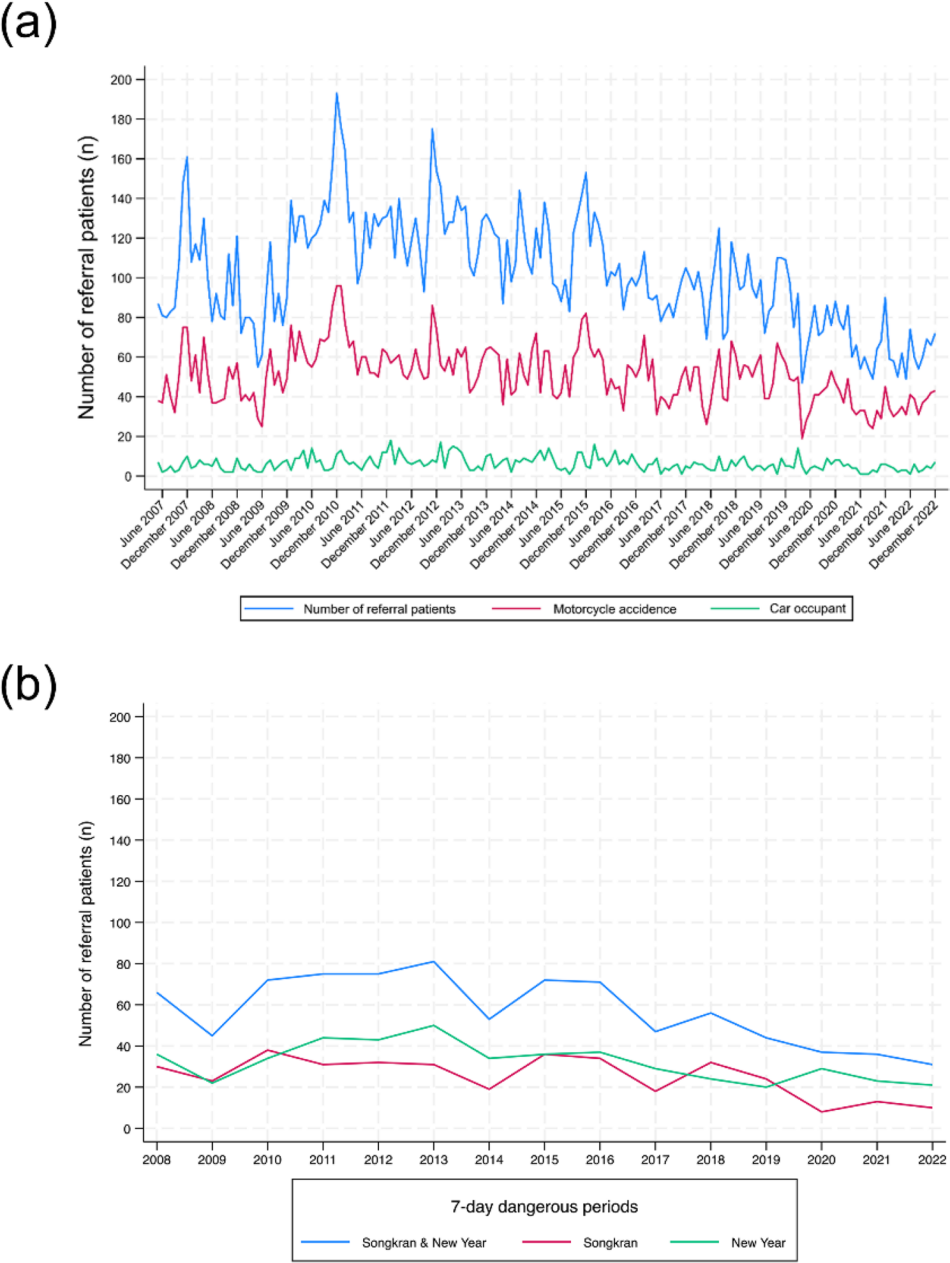
Number of referred patients: (**a**) overall and from motorcycle and car crashes, and (**b**) during the “Seven Dangerous Days” periods

Crash-related referrals showed a decreasing trend over the study period (Fig. 1a). The referral frequency displayed seasonality: higher during June–December, lower during January–May, and peaking in December 2010. Furthermore, referrals from motorcycle crashes followed the same trend, while referrals from car crashes varied over time (Fig. 1a). ADF testing of referred patient data showed that the latter were non-stationary (*p* = 0.36) (See Supplementary Fig. S5a). However, first differencing transformation of the data caused them to become stationary (*p* < 0.001) (See Supplementary Fig. S5(b)). This was also the case for motorcycle crash referrals (Supplementary Fig. S6) and car crash referrals (See Supplementary Fig. S7).

According to the entries in the supplementary Table S2, for all of the referred patients, the SARIMA (1,1,1)(1,0,1)_12_ model attained the lowest AIC, BIC, and RMSE values (1312.47, 1318.56, and 17.11, respectively), thereby supporting the notion that it fits the data well. In addition, the Ljung-Box Q test results indicate that the model did not display any substantial lack of fit (*p* = 0.307). Furthermore, the SARIMA(0,1,2)(1,0,1)_12_ attained the lowest AIC and BIC values for analyzing both motorcycle-crash referrals (1160.87 and 1166.94, respectively) and car crash referrals (835.35 and 841.42, respectively). Based on these results, we selected the SARIMA (1,1,1)(1,0,1)_12_ model to predict the overall number of referrals and the SARIMA (0,1,2)(1,0,1) _12_ model to predict the numbers of motorcycle-crash and car-crash referrals.

**Fig. 2:**
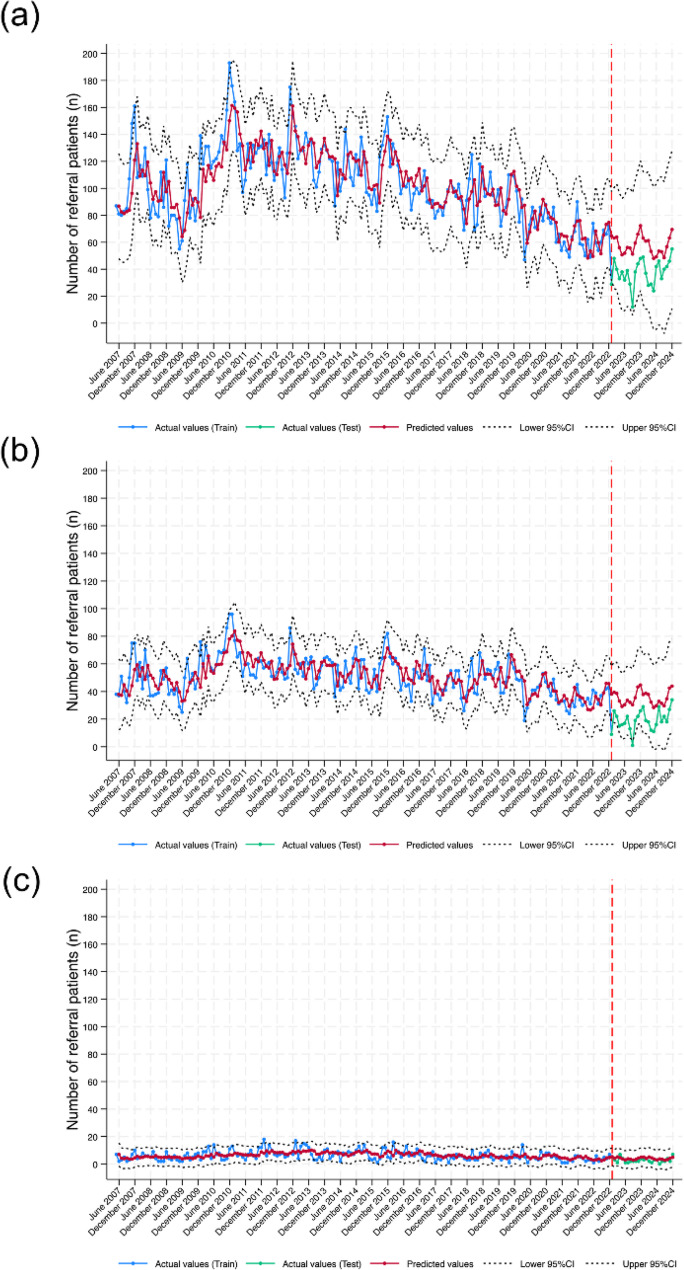
Predict Number of referred patients (**a**) overall referral patients, (**b**) motorcycle crashes, and (**c**) car crashes

Table 2 reports the estimated parameter values and performance metrics for the SARIMA models used to predict the overall number of referrals and those from motorcycle and car crashes. The MAE and RMSE values for the overall patient referrals model are 20.428 and 21.971, respectively. The MAPE value (representing the mean percentage difference between the actual and predicted values) is 67.071%, while the PI Cov.% value for the actual value is 95.83%. These closely aligned with the observed values, as shown in Fig. 2. The predicted value for car crash referrals provided the narrowest 95% confidence interval (CI) (Fig. 2c), thereby inferring good predictive performance.

**Table 2:** Estimated parameters for the SARIMA models and forecast evaluation metrics for predicting patient referrals

Model Parameter	Estimate	Standard Error	*P*-value	95%CI		MAE	MAPE%	RMSE	PI Cov.%
				Lower	Upper				
Total patient referrals: SARIMA(1,1,1)(1,0,1)_12_						20.428	67.071	21.971	95.83
Constant	-0.206	0.653	0.752	-1.486	1.074				
AR (1)	0.363	0.106	< 0.001	0.155	0.571				
MA (1)	0.825	0.065	< 0.001	0.698	0.952				
AR (1) seasonal	0.995	0.045	< 0.001	0.907	1.083				
MA (1) seasonal	0.959	0.204	< 0.001	0.559	1.359				
Referrals from motorcycle crashes: SARIMA(0,1,2)(1,0,1)_12_						16.152	212.092	17.164	91.67
Constant	-0.061	0.449	0.892	-0.941	0.819				
MA (1)	0.568	0.074	< 0.001	0.423	0.713				
MA (2)	0.163	0.074	0.029	0.018	0.308				
AR (1) seasonal	1.000	0.006	< 0.001	0.988	1.012				
MA (1) seasonal	0.984	0.092	< 0.001	0.804	1.164				
Referrals from car crashes: SARIMA(0,1,2)(1,0,1)_12_						1.702	91.394	2.060	100.00
Constant	-0.006	0.036	0.868	-0.077	0.065				
MA (1)	0.808	0.074	< 0.001	0.663	0.953				
MA (2)	0.098	0.074	0.186	-0.047	0.243				
AR (1) seasonal	0.996	0.075	< 0.001	0.849	1.143				
MA (1) seasonal	0.970	0.263	< 0.001	0.455	1.485				

95% CI: 95% confidence interval, *AR* autoregressive, *MA *moving average, *SARIMA *Seasonal autoregressive integrated moving average, *MAE *mean absolute error, *MAPE*% mean absolute percentage error, *RMSE *root mean square error, *PI Cov*.%: predicted interval coverage at the 95% confidence level

### Sensitivity analyses for the total number of referrals

**Fig. 3:**
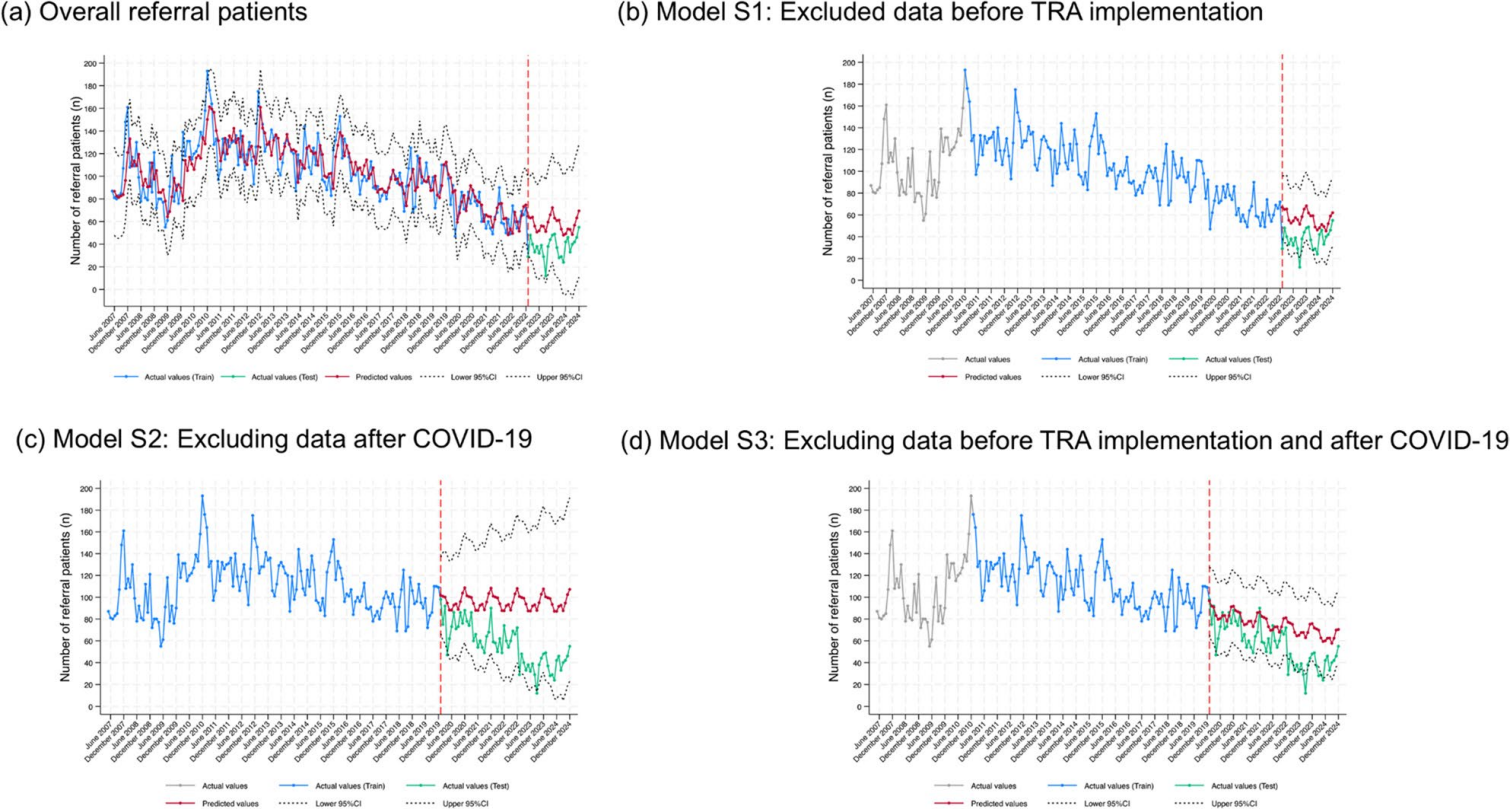
Sensitivity analysis models for examining the impact of TRA implementation and the COVID-19 pandemic on total referrals prediction accuracy, (**a**) Overall referral patients, (**b**) Model S1: Excluding data before TRA implementation, (**c**) Model S2: Excluding data after COVID-19, and (**d**) Model S3: Excluding data before TRA implementation and after COVID-19

The results for the sensitivity analysis model excluding entries before full TRA implementation (January 2007 to December 2010) from the training dataset in Supplementary Table S4, Fig. 3b and Table 3 show predicted values closest to the actual values for patient referrals between January 2023 to December 2024 (MAE = 18.958, MAPE = 63.394%, RMSE = 20.993, and PI Cov.% = 87.50%). The results for the sensitivity analysis model after excluding entries during the COVID-19 pandemic period in Thailand (January 2019 to December 2022) from the training dataset in Fig. 3c and Table 3 indicate that the predicted values did not agree with the actual values (MAE = 39.750, MAPE = 97.742%, RMSE = 43.617, and PI Cov.% = 95.00%); the confidence interval for the estimation was also larger than that for the baseline model. The sensitivity model results in which entries from before TRA implementation and after the COVID-19 pandemic were excluded from the training dataset are shown in Fig. 3d; although the CI for the predicted value was narrower than that for the baseline model (MAE = 22.348, MAPE = 60.880%, and RMSE = 13.121), its PI Cov.% was lower than that for Model S1 (81.25%) (Table 3).

**Table 3:** Forecast evaluation metrics for the sensitivity analysis

Period	MAE	MAPE%	RMSE	PI Cov.%
Total				
Overall period	20.428	67.071	21.971	95.83
After TRA implementation	18.958	63.394	20.993	87.50
Before the COVID-19 pandemic	39.750	97.742	43.617	95.00
After TRA implementation and before the COVID-19 pandemic	22.348	60.880	13.121	81.25

*MAE* mean absolute error, *MAPE*% mean absolute percentage error, *RMSE *root mean square error, *PI Cov*.% predicted interval coverage at the 95% confidence level

### The “Seven dangerous Days” periods

The trend in crash-related referrals during Songkran and the New Year festivals has been decreasing since 2019 (Fig. 1b). The majority of referrals occurred within Chiang Mai each year, and the peak time for patient referrals occurred between 18:00 and 23:59 each year except for 2009 and 2020 (See Supplementary Fig. S8). In contrast, crash referrals during the New Year period have increased since 2019 (See Supplementary Fig. S9). The referred patient data during the New Year period were found to be non-stationary (ADF test: *p* = 0.697), which became stationary after performing first differencing transformation (*p* < 0.001) (Supplementary Fig. S10). A similar pattern was observed for the Songkran period (*p* = 0.963 and *p* < 0.001 before and after first differencing transformation, respectively) (See Supplementary Fig. S11).

**Fig. 4:**
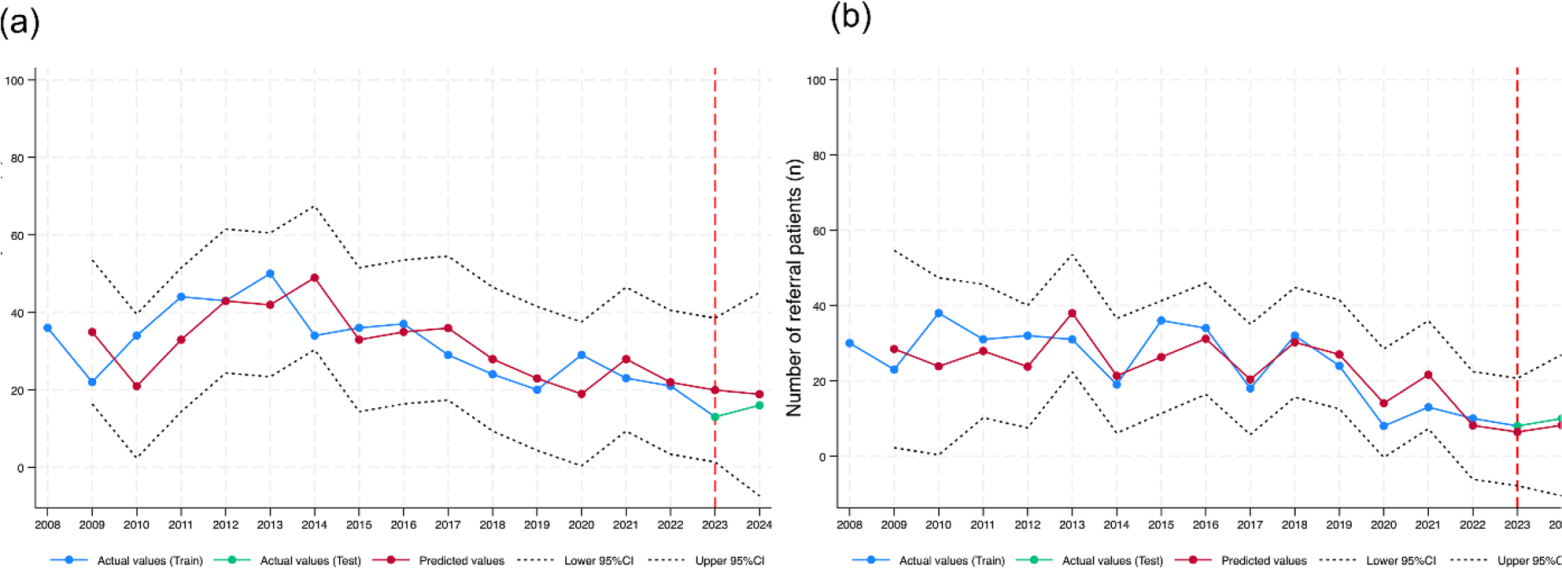
Predict Number of referred patients on seven dangerous days periods (**a**) New year period and (**b**) Songkran period

As reported in the supplementary Table S3, the ARIMA(0,1,0) model showed the best fit for the New Year period data (AIC = 102.93, BIC = 104.2, and RMSE = 8.6). Moreover, the Ljung-Box Q test results show that the model does not display a substantial lack of fit (*p* = 0.426). Following the same model selection criteria, the ARIMA(2,1,1) model was identified as the most suitable for the Songkran period. Table 4 provides the estimated parameter values for the ARIMA models and forecast evaluation metrics for the two “Seven Dangerous Days” periods covering the New Year and Songkran. Both models demonstrated low error metric values, with MAE and RMSE values of 4.893 and 5.299, and 1.693 and 1.639, respectively. Furthermore, the PI Cov.% reached 100% for both periods. Figure 4a illustrates that the observed and predicted numbers of referred patients during the New Year period were within the 95% CI. In addition, the predicted numbers of referred patients during the Songkran period closely aligned with the observed numbers since 2016 (Fig. 4b).

**Table 4:** Estimated parameters for the ARIMA model and forecast evaluation metrics for the “Seven dangerous Days” periods

Model Parameter	Estimate	Standard Error	*P*-value	95%CI		MAE	MAPE%	RMSE	PI Cov.%
				Lower	Upper				
New Year: ARIMA(0,1,0)						4.893	35.577	5.299	100.00
Constant	-1.071	2.298	0.649	-5.575	3.433				
Songkran: ARIMA(2,1,1)						1.693	18.916	1.696	100.00
Constant	-1.556	1.197	0.223	-3.902	0.790				
AR (1)	-1.074	0.181	< 0.001	-1.429	-0.719				
AR (2)	-0.809	0.156	< 0.001	-1.115	-0.503				
MA (1)	-0.990	8.512	0.910	-17.674	15.694				

95% *CI* 95% confidence interval, *AR *autoregressive, *MA *moving average, *ARIMA *Autoregressive Integrated Moving Average, *MAE *mean absolute error, *MAPE*% mean absolute percentage error, *RMSE *root mean square error, *PI Cov*.% predicted interval coverage at the 95% confidence level

## Discussion

This study was conducted on data from the records of 19,164 patients involved in crashes who were referred to Maharaj Nakorn Chiang Mai Hospital between May 2007 and December 2022 to provide time-series models for predicting the number of referred patients. The data show that nearly half of the referred patients (49.8%) had been involved in motorcycle crashes, while car crashes accounted for 6.1%.

Based on these data, we applied a time-series analysis to predict the total number of crash-related referrals to Maharaj Nakorn Chiang Mai Hospital, as well as specifically those from accidents involving motorcycles or cars. As the data appeared to display a seasonal pattern, comparative analyses were performed using both the ARIMA and SARIMA approaches. Our results indicate that the SARIMA (1,1,1)(1,0,1)_12_ model was appropriate for forecasting the total number of the referred patients, while the SARIMA (0,1,2)(1,0,1)_12_ model was suitable for predicting the numbers of referrals from motorcycle and car crashes. Although model evaluation using MAE and RMSE showed acceptable error levels for both models, the MAPE for the motorcycle crashes referrals model was noticeably high (212%), which is largely due to very few referrals occurring during some months. Thereby, the small denominators made the MAPE value disproportionately large, thus indicating that this performance metric may not be the most suitable model-performance indicator for these specific periods. On the other hand, the PI Cov.% results were satisfactory, with values exceeding 90% across all categories (95.8%, 91.67%, and 100% for total referrals and those from motorcycle and car crashes, respectively). This demonstrates that the prediction intervals constructed by the models were wide enough to encompass the actual patient numbers in most scenarios. Our forecasting analysis revealed a rising trend in the number of motorcycle crashes-related referrals. However, our results differ from those of Jomnonkwao [22], who suggested that ARIMA models are not appropriate for forecasting the number of traffic accident-related referrals and employed a time-series model using exponential smoothing instead. This divergence may be due to differences in data characteristics: their study predicted deaths nationwide, while the current study predicted the number of referrals to a single hospital, along with variations in the study location and timeframe. Conversely, our findings are consistent with a study on time-series-based prediction of road crash-related injuries in Kurdistan [23]; the authors reported that the SARIMA (1,0,2)(1,0,0)_12_ model is suitable for forecasting motorcycle-crash referrals. Moreover, these findings, along with ours, suggest that a decrease in motorcycle helmet usage (Supplementary Fig. S4(b)) is related to an increase in crash-related referrals to ED departments. The ability to forecast patient referrals using the SARIMA model allows for proactive resource management. The hospital administrators can use our model to anticipate demand and allocate staff and medical supplies well in advance.

According to the results of the sensitivity analysis, the predicted values using Model S1 were closer to the actual values, with narrower CIs and higher PI coverage than the baseline and other sensitivity models. These results suggest that considering the impact of full TRA implementation enhanced the prediction accuracy. As reported in our previous study [21], TRA implementation could have helped to reduce patient overcrowding in emergency departments.

Surprisingly, excluding data during the COVID-19 pandemic worsened the prediction accuracy. This might be related to fewer accidents and referrals occurring during the government lockdown period and changes in the referral policy before and after the lockdown. However, we could not examine the impact of policy changes during COVID-19 because of the short period for the training dataset. Future studies should consider the impact of this potentially confounding factor.

Our findings suggest that the SARIMA-based patient referral forecasting model, for which we considered structural changes during the study period, could provide useful information for simulations toward proactive hospital management. By applying this model, administrators can accurately anticipate patient demand and strategically plan staffing levels and medical supply allocation in advance, especially in response to policy changes or new pandemics, leading to more efficient and responsive healthcare delivery.

When examining the mortality rates according to risk behaviors, that for patients who did not wear a helmet was higher than for those who did. This result reflects the importance of wearing this protective device to reduce the severity of trauma and the likelihood of death from traffic accidents involving motorcycles. Advocating for the use of this protective device is a key factor in campaigns for road safety in Thailand.

The high volume of domestic and international tourists visiting Chiang Mai during the New Year and Songkran festivals is accompanied by a significant increase in the number of road users and, thus, crashes during the “Seven Dangerous Days” periods. With this in mind, our time-series analysis revealed that ARIMA (0,1,0) and ARIMA (2,1,1) models were the most suitable for predicting the number of crash-related referrals during the New Year and Songkran periods, respectively. The models demonstrated a high degree of reliability (as evidenced by PI Cov.% values of 100% across both periods), which indicates that the observed values remained entirely within the predicted boundaries. Although their MAPE values exceeded 10% (35.58% for the New Year period and 18.92% for the Songkran period), likely due to the limited two-year dataset and sharp seasonal fluctuations, their relatively low MAE and RMSE values suggest that the models are robust and practical for aiding decision-making during these volatile periods.

Our findings showed good agreement between the observed and forecasted values, particularly during the Songkran festival. The data for patient referrals during the “Seven Dangerous Days” periods has remained relatively stable over time, likely due to consistent law enforcement and public campaigns aimed at mitigating crashes, which contrasts with the more variable data during other times of the year. Nevertheless, there has been a decreasing trend in crash-related referrals in recent years, possibly because only severe cases (ISS > 9) are being transferred to Maharaj Nakorn Chiang Mai Hospital for treatment [21].

The strength of this study is its use of a substantial volume of long-term data, which is suitable for time-series modeling. This extensive dataset enabled clear identification of underlying trends and seasonal patterns due to the reliability of the data from a hospital with a well-established patient referral system and inter-hospital collaboration [21]. In addition, our unique focus on Thailand’s “Seven Dangerous Days” periods addresses a critical area of policy concern due to the historically high incidence of crashes during these periods. Despite its strengths, this study has several limitations. The findings are specific to the context of Chiang Mai and may not be generalizable to other areas with different social and geographical contexts. Therefore, future studies should consider expanding the scope to other regions. Although we examined the mortality rate trends according to risk behavior, several important predictors of crash severity, such as the mechanism, protective device use, alcohol/timing/context, road type, vehicle speed proxies, and referral patterns, such as prehospital care and injury descriptors, were not included in the study. Further investigations should adjust for these factors. Moreover, the high proportion of missingness in the data for some of the behavioral variables (29.73% for alcohol consumption, 28.37% for helmet use in motorcycle crashes, and 40.69% for seatbelt use in car crashes), the mortality rate trend reported in this study might not be appropriate for generalizing the mortality rate for traffic crashes. Finally, the sensitivity analysis to evaluate the robustness of the prediction model in response to structural changes was focused on only major changes caused by TRA implementation and the COVID-19 pandemic. Other minor changes should be considered in future investigations.

## Conclusions

The SARIMA model was appropriate for forecasting monthly patient referrals in our study settings, especially for car crashes, while the ARIMA model demonstrated good predictive performance for the “Seven Dangerous Days” periods, particularly during the Songkran festival. Including and excluding data from TRA implementation and the COVID-19 pandemic affected the prediction performances. Utilizing these models after adjusting for these major events could assist hospitals and relevant agencies in high-risk areas or during festival periods by providing predictions of traffic accident-related referrals that would enable the proactive allocation of necessary staff and resources.

## Supplementary Information

Supplementary Material 1.

## Data Availability

The datasets used and/or analyzed during the current study are available from the corresponding author on reasonable request.

## Abbreviations

ACF: Autocorrelation function
AIC: Akaike information criterion
ADF: Augmented dickey-fuller
AR: Autoregressive
ARIMA: Autoregressive integrated moving average
ATLS: Advanced trauma life support
BIC: Bayesian information criterion
CI: Confidence interval
EDs: Emergency departments
EKG: Electrocardiogram
IQR: Interquartile range
ISS: Injury severity score
MA: Moving average
MAE: Mean absolute error
MAPE: Mean absolute percentage error
PACF: Partial Autocorrelation Function
PI Cov.%: Prediction interval coverage at 95%
RMSE: Root mean square error
SARIMA: Seasonal ARIMA
WHO: World Health Organization
