# Supplementary material for: Time-series-based forecasting of accident-related referrals to Maharaj Nakorn Chiang Mai Hospital, Northern Thailand, during each year and especially the “Seven dangerous Days” periods
Source: BMC Public Health. 2026 Jan 17;26:572. doi: 10.1186/s12889-026-26304-9 (PMC12895657; doi:10.1186/s12889-026-26304-9)
Supplement: Supplementary file 1 — Supplementary Material 1. [file 12889_2026_26304_MOESM1_ESM.docx]

**Supplementary Table S1.** “Seven dangerous days” periods for the New year and Songkran.

| **Year** | **New year** | **Songkran** |
| --- | --- | --- |
| 2007 | December 28, 2006, to January 3, 2007 | April 11–17, 2007 |
| 2008 | December 28, 2007, to January 3, 2008 | April 11–17, 2008 |
| 2009 | December 30, 2008, to January 5, 2009 | April 11–17, 2009 |
| 2010 | December 29, 2009, to January 4, 2010 | April 11–17, 2010 |
| 2011 | December 29, 2010, to January 4, 2011 | April 11–17, 2011 |
| 2012 | December 29, 2011, to January 4, 2012 | April 11–17, 2012 |
| 2013 | December 27, 2012, to January 2, 2013 | April 11–17, 2013 |
| 2014 | December 27, 2013, to January 2, 2014 | April 11–17, 2014 |
| 2015 | December 30, 2014, to January 5, 2015 | April 11–17, 2015 |
| 2016 | December 29, 2015, to January 4, 2016 | April 11–17, 2016 |
| 2017 | December 29, 2016, to January 4, 2017 | April 11–17, 2017 |
| 2018 | December 28, 2017, to January 3, 2018 | April 11–17, 2018 |
| 2019 | December 27, 2018, to January 2, 2019 | April 11–17, 2019 |
| 2020 | December 27, 2019, to January 1, 2020 | April 11–17, 2020 |
| 2021 | December 29, 2020, to January 4, 2021 | April 11–17, 2021 |
| 2022 | December 29, 2021, to January 4, 2022 | April 11–17, 2022 |

## **Supplementary Table S2.** Comparison of model candidates of the referred patients by ARIMA and SARIMA models.

|  | **AIC** | **BIC** | **RMSE** | **Ljung-Box Q test** | |
| --- | --- | --- | --- | --- | --- |
|  |  |  |  | **Q statistics** | **p-value** |
| **Model: overall referred patients** | | | | | |
| **ARIMA (d=1)** |  |  |  |  |  |
| p=1, q=1 | 1606.90 | 1619.80 | 17.53 | 19.01 | 0.268 |
| p=2, q=1 | 1608.50 | 1624.60 | 17.57 | 18.51 | 0.237 |
| p=1, q=2 | 1608.80 | 1624.90 | 17.57 | 18.53 | 0.236 |
| p=2, q=2 | 1610.60 | 1630.00 | 17.61 | 18.71 | 0.176 |
| p=0, q=2 | 1612.80 | 1625.80 | 17.80 | 26.47 | 0.048 |
| p=0, q=1 | 1619.60 | 1629.30 | 18.18 | 30.36 | 0.024 |
| p=2, q=0 | 1624.10 | 1637.10 | 18.36 | 27.08 | 0.041 |
| p=1, q=0 | 1626.40 | 1636.10 | 18.52 | 30.32 | 0.024 |
| p=0, q=0 | 1637.80 | 1644.30 | 19.15 | 37.71 | 0.004 |
| **SARIMA** |  |  |  |  |  |
| (0,1,0)(0,0,1)_12_ | 1637.26 | 1646.95 | 19.07 | 36.03 | 0.005 |
| (0,1,0)(1,0,0)_12_ | 1637.10 | 1646.79 | 19.07 | 36.30 | 0.004 |
| (0,1,0)(1,0,1)_12_ | 1635.81 | 1639.04 | 18.90 | 40.06 | <0.001 |
| (0,1,1)(0,0,1)_12_ | 1618.77 | 1631.70 | 18.10 | 25.72 | 0.058 |
| (0,1,1)(1,0,0)_12_ | 1618.28 | 1631.21 | 18.08 | 25.46 | 0.062 |
| (0,1,1)(1,0,1)_12_ | 1605.68 | 1621.83 | 17.47 | 23.55 | 0.073 |
| (0,1,2)(0,0,1)_12_ | 1612.24 | 1628.40 | 17.73 | 21.03 | 0.136 |
| (0,1,2)(1,0,0)_12_ | 1611.80 | 1627.95 | 17.71 | 20.48 | 0.154 |
| (0,1,2)(1,0,1)_12_ | 1600.81 | 1620.20 | 17.21 | 17.35 | 0.238 |
| (1,1,0)(0,0,1)_12_ | 1625.69 | 1638.61 | 18.44 | 26.64 | 0.046 |
| (1,1,0)(1,0,0)_12_ | 1625.36 | 1638.29 | 18.43 | 26.58 | 0.046 |
| (1,1,0)(1,0,1)_12_ | 1614.59 | 1617.81 | 18.02 | 26.69 | 0.031 |
| (1,1,1)(0,0,1)_12_ | 1606.09 | 1622.24 | 17.46 | 16.62 | 0.342 |
| (1,1,1)(1,0,0)_12_ | 1605.77 | 1621.93 | 17.45 | 16.76 | 0.333 |
| **(1,1,1)(1,0,1)_12_** | **1312.47** | **1318.56** | **17.11** | **16.10** | **0.307** |
| (1,1,2)(0,0,1)_12_ | 1607.74 | 1627.13 | 17.49 | 15.61 | 0.338 |
| (1,1,2)(1,0,0)_12_ | 1607.38 | 1626.76 | 17.48 | 15.64 | 0.336 |
| (1,1,2)(1,0,1)_12_ | 1598.14 | 1617.53 | 17.14 | 15.31 | 0.288 |
| **Model: referred patients involved in motorcycle crashes** | | | | | |
| **ARIMA** |  |  |  |  |  |
| **p=1, q=1** | **1430.14** | **1443.07** | **10.94** | 22.96 | 0.115 |
| p=2, q=1 | 1432.09 | 1448.24 | 10.97 | 23.22 | 0.080 |
| p=1, q=2 | 1432.06 | 1448.22 | 10.97 | 23.35 | 0.077 |
| p=2, q=2 | 1433.71 | 1453.09 | 10.99 | 22.99 | 0.060 |
| p=0, q=2 | 1434.49 | 1447.41 | 11.06 | 32.80 | 0.008 |
| p=0, q=1 | 1445.02 | 1454.71 | 11.40 | 46.79 | <0.001 |
| p=2, q=0 | 1448.65 | 1461.57 | 11.48 | 33.97 | 0.005 |
| p=1, q=0 | 1457.86 | 1467.55 | 11.80 | 41.56 | <0.001 |
| p=0, q=0 | 1470.29 | 1476.76 | 12.24 | 38.12 | <0.001 |
| **SARIMA** |  |  |  |  |  |
| (0,1,0)(0,0,1)_12_ | 1468.48 | 1478.17 | 12.15 | 31.03 | 0.020 |
| (0,1,0)(1,0,0)_12_ | 1467.75 | 1477.45 | 12.13 | 30.75 | 0.021 |
| (0,1,0)(1,0,1)_12_ | 1464.72 | 1477.65 | 12.02 | 34.51 | 0.005 |
| (0,1,1)(0,0,1)_12_ | 1441.01 | 1453.93 | 11.26 | 29.25 | 0.022 |
| (0,1,1)(1,0,0)_12_ | 1438.89 | 1451.81 | 11.20 | 25.64 | 0.059 |
| (0,1,1)(1,0,1)_12_ | 1168.72 | 1171.76 | 10.66 | 16.08 | 0.377 |
| (0,1,2)(0,0,1)_12_ | 1431.70 | 1447.85 | 10.95 | 19.70 | 0.184 |
| (0,1,2)(1,0,0)_12_ | 1430.40 | 1446.56 | 10.92 | 17.15 | 0.310 |
| **(0,1,2)(1,0,1**)_12_ | **1160.87** | **1166.94** | **10.54** | **10.72** | **0.708** |
| (1,1,0)(0,0,1)_12_ | 1455.27 | 1468.19 | 11.70 | 30.77 | 0.014 |
| (1,1,0)(1,0,0)_12_ | 1454.04 | 1466.97 | 11.66 | 29.87 | 0.019 |
| (1,1,0)(1,0,1)_12_ | 1442.79 | 1458.94 | 11.34 | 30.53 | 0.010 |
| (1,1,1)(0,0,1)_12_ | 1427.38 | 1443.53 | 10.84 | 14.02 | 0.524 |
| (1,1,1)(1,0,0)_12_ | 1426.32 | 1442.48 | 10.82 | 13.41 | 0.571 |
| (1,1,1)(1,0,1)_12_ | 1168.58 | 1174.67 | 10.54 | 10.97 | 0.688 |
| (1,1,2)(0,0,1)_12_ | 1429.07 | 1448.46 | 10.86 | 14.12 | 0.441 |
| (1,1,2)(1,0,0)_12_ | 1427.80 | 1447.19 | 10.83 | 13.47 | 0.490 |
| (1,1,2)(1,0,1)_12_ | 1415.12 | 1437.73 | 10.56 | 10.88 | 0.621 |
| **Model: referred patients involved in car crashes** | | | | | |
| **ARIMA (d=1)** |  |  |  |  |  |
| **p=1, q=1** | **984.07** | **996.99** | **3.31** | 15.63 | 0.479 |
| p=2, q=1 | 985.53 | 1001.68 | 3.32 | 15.63 | 0.407 |
| p=1, q=2 | 985.54 | 1001.69 | 3.32 | 15.45 | 0.420 |
| p=2, q=2 | 987.04 | 1006.43 | 3.32 | 15.38 | 0.353 |
| p=0, q=2 | 984.68 | 997.61 | 3.32 | 16.61 | 0.411 |
| p=0, q=1 | 986.79 | 996.49 | 3.35 | 25.09 | 0.093 |
| p=2, q=0 | 1015.96 | 1028.88 | 3.61 | 27.87 | 0.033 |
| p=1, q=0 | 1027.28 | 1036.97 | 3.73 | 40.87 | <0.001 |
| p=0, q=0 | 1065.37 | 1071.84 | 4.14 | 52.30 | <0.001 |
| **SARIMA** |  |  |  |  |  |
| (0,1,0)(0,0,1)_12_ | 1065.43 | 1075.12 | 4.13 | 51.14 | <0.001 |
| (0,1,0)(1,0,0)_12_ | 1065.33 | 1075.03 | 4.13 | 51.30 | <0.001 |
| (0,1,0)(1,0,1)_12_ | 1067.25 | 1080.17 | 4.14 | 51.42 | <0.001 |
| (0,1,1)(0,0,1)_12_ | 986.12 | 999.05 | 3.33 | 18.63 | 0.288 |
| (0,1,1)(1,0,0)_12_ | 985.66 | 998.58 | 3.33 | 17.96 | 0.326 |
| (0,1,1)(1,0,1)_12_ | 839.64 | 842.69 | 3.30 | 11.08 | 0.747 |
| (0,1,2)(0,0,1)_12_ | 983.96 | 1000.11 | 3.30 | 12.02 | 0.678 |
| (0,1,2)(1,0,0)_12_ | 983.55 | 999.71 | 3.30 | 11.75 | 0.698 |
| **(0,1,2)(1,0,1)_12_** | **835.35** | **841.42** | **3.29** | **8.80** | **0.844** |
| (1,1,0)(0,0,1)_12_ | 1026.53 | 1039.46 | 3.72 | 35.60 | 0.003 |
| (1,1,0)(1,0,0)_12_ | 1026.19 | 1039.11 | 3.71 | 35.48 | 0.003 |
| (1,1,0)(1,0,1)_12_ | 1027.14 | 1043.30 | 3.71 | 35.20 | 0.002 |
| (1,1,1)(0,0,1)_12_ | 983.26 | 999.41 | 3.30 | 11.14 | 0.743 |
| (1,1,1)(1,0,0)_12_ | 982.85 | 999.01 | 3.29 | 10.93 | 0.758 |
| (1,1,1)(1,0,1)_12_ | 982.17 | 1001.56 | 3.29 | 8.54 | 0.859 |
| (1,1,2)(0,0,1)_12_ | 984.49 | 1003.88 | 3.30 | 10.42 | 0.731 |
| (1,1,2)(1,0,0)_12_ | 984.03 | 1003.42 | 3.30 | 10.21 | 0.747 |
| (1,1,2)(1,0,1)_12_ | 983.61 | 1006.23 | 3.29 | 7.94 | 0.848 |

ARIMA: autoregressive integrated moving average, SARIMA: Seasonal autoregressive integrated moving average, AIC: Akaike information criterion, BIC: Bayesian Information Criterion, RMSE: root mean square error.

## **Supplementary Table S3.** Comparison of model candidates of the referred patients during seven dangerous days periods

| **Model (d=1)** | **AIC** | **BIC** | **RMSE** | **Ljung-Box Q test** | |
| --- | --- | --- | --- | --- | --- |
|  |  |  |  | **Q statistics** | **p-value** |
| **Model: seven dangerous days during New Year period** | | | | | |
| p=1, q=1 | 104.08 | 106.64 | 8.65 | 8.22 | 0.768 |
| p=2, q=1 | 106.03 | 109.22 | 9.07 | 9.16 | 0.689 |
| p=1, q=2 | 103.28 | 105.84 | 9.07 | 9.47 | 0.662 |
| p=2, q=2 | 106.55 | 110.39 | 9.21 | 9.64 | 0.648 |
| p=0, q=2 | 104.15 | 106.71 | 8.75 | 11.39 | 0.496 |
| p=0, q=1 | 103.61 | 105.52 | **8.60** | 8.00 | 0.785 |
| p=2, q=0 | 105.60 | 108.15 | 8.97 | 8.55 | 0.741 |
| p=1, q=0 | 103.97 | 105.88 | 8.68 | 9.24 | 0.683 |
| **p=0, q=0** | **102.93** | **104.21** | **8.60** | **12.25** | **0.426** |
| **Model: seven dangerous days during Songkran period** | | | | | |
| p=1, q=1 | 110.13 | 112.68 | 9.19 | 15.95 | 0.194 |
| **p=2, q=1** | **100.54** | **103.73** | **7.72** | **8.50** | **0.745** |
| p=1, q=2 | 103.82 | 106.37 | 8.96 | 12.84 | 0.381 |
| p=2, q=2 | 100.54 | 103.74 | 8.14 | 8.45 | 0.749 |
| p=0, q=2 | 107.16 | 109.71 | 9.20 | 18.83 | 0.093 |
| p=0, q=1 | 111.57 | 113.49 | 8.80 | 24.49 | 0.017 |
| p=2, q=0 | 103.78 | 106.34 | 8.51 | 8.32 | 0.760 |
| p=1, q=0 | 108.10 | 110.02 | 9.97 | 15.89 | 0.196 |
| p=0, q=0 | 109.53 | 110.81 | 10.88 | 24.44 | 0.018 |

AIC: Akaike information criterion, BIC: Bayesian Information Criterion, RMSE: root mean square error.

**Supplementary Table S4.** Estimated parameters for sensitivity analysis

| **Model parameter** | **Estimate** | **Standard error** | ***P*-value** | **95%CI** | |
| --- | --- | --- | --- | --- | --- |
|  |  |  |  | **Lower** | **Upper** |
| Model S1: SARIMA(1,1,2)(1,0,1)12 | | | | | |
| Constant | -0.527 | 0.058 | <0.001 | -0.641 | -0.413 |
| AR (1) | 0.409 | 0.225 | 0.071 | -0.032 | 0.850 |
| MA (1) | 1.036 | 0.281 | <0.001 | 0.485 | 1.587 |
| MA (2) | -0.037 | 0.235 | 0.874 | -0.498 | 0.424 |
| AR (1) seasonal | 0.998 | 0.070 | <0.001 | 0.861 | 1.135 |
| MA (1) seasonal | 0.969 | 0.430 | 0.026 | 0.126 | 1.812 |
| Model S2: SARIMA(1,1,1)(1,0,1)12 | | | | | |
| Constant | -0.019 | 0.591 | 0.974 | -1.177 | 1.139 |
| AR (1) | 0.430 | 0.110 | <0.001 | 0.214 | 0.646 |
| MA (1) | 0.861 | 0.063 | <0.001 | 0.738 | 0.984 |
| AR (1) seasonal | 0.996 | 0.082 | <0.001 | 0.835 | 1.157 |
| MA (1) seasonal | 0.964 | 0.342 | 0.005 | 0.294 | 1.634 |
| Model S3: SARIMA(0,1,2)(1,0,1)12 | | | | | |
| Constant | -0.434 | 0.079 | <0.001 | -0.589 | -0.279 |
| MA (1) | 0.615 | 1.016 | 0.547 | -1.376 | 2.606 |
| MA (2) | 0.385 | 0.417 | 0.358 | -0.432 | 1.202 |
| AR (1) seasonal | 0.983 | 0.184 | <0.001 | 0.622 | 1.344 |
| MA (1) seasonal | 0.927 | 0.433 | 0.035 | 0.078 | 1.776 |

95%CI: 95% confident interval, AR: autoregressive, MA: moving average, SARIMA: Seasonal autoregressive integrated moving average, Model S1: Excluded data before TRA implementation, Model S2: Excluded data after COVID-19, Model S3: Excluded data before TRA implementation and after COVID-19


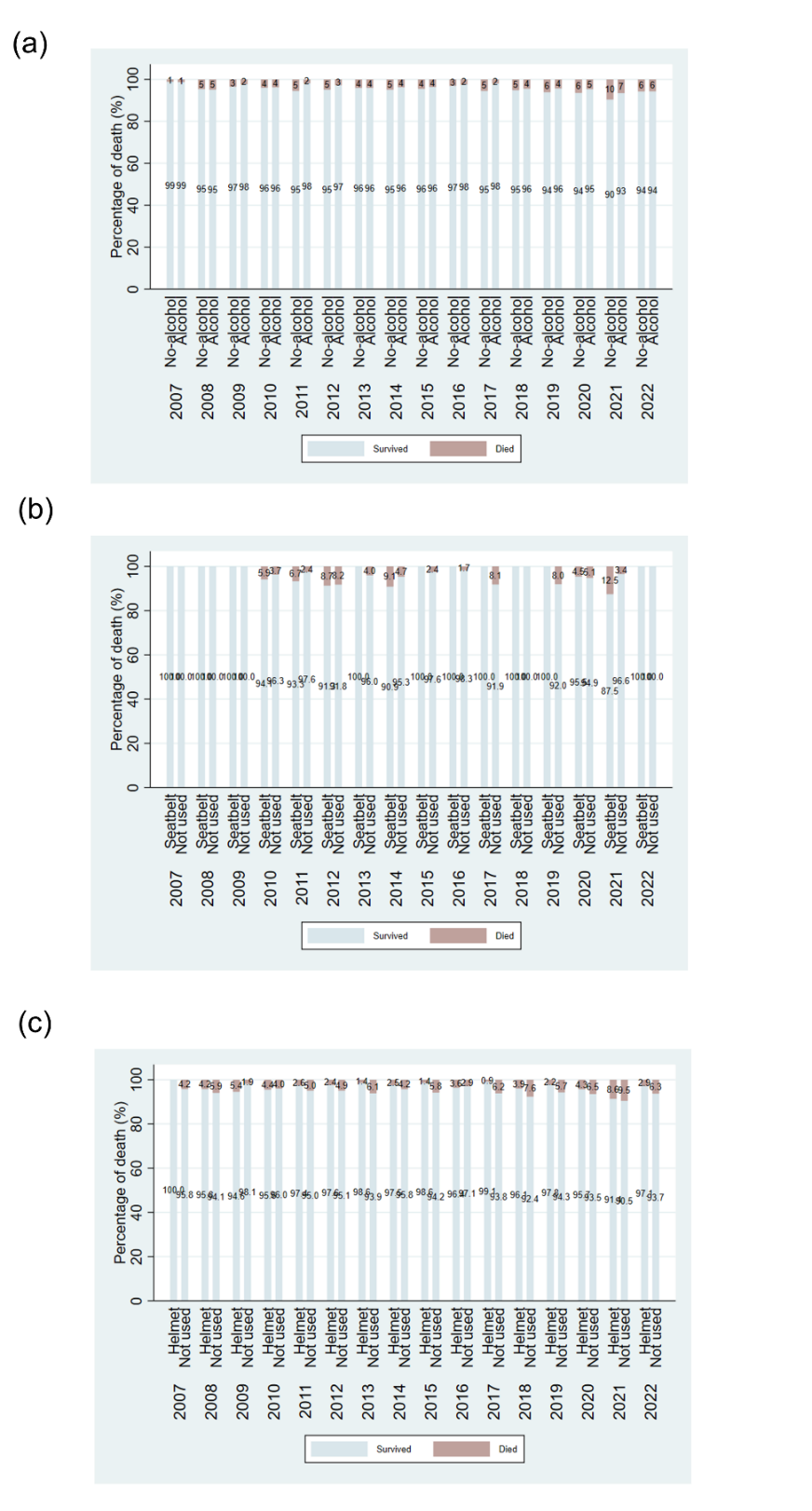


**Supplementary Fig. S1** Mortality rates for patients referred to Maharaj Nakorn Chiang Mai Hospital between 2007 and 2022 by risk behavior: (a) alcohol consumption, (b) not using a seatbelt, and (c) not wearing a helmet

| 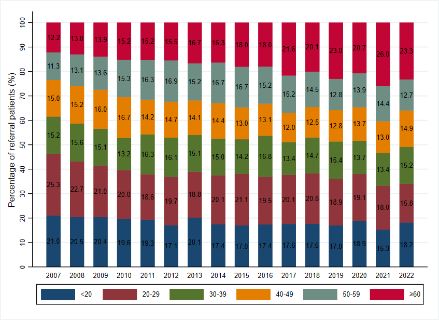  (a) | 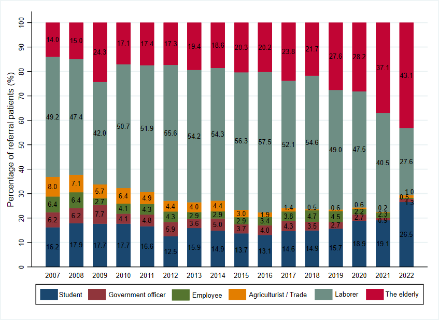  (b) |
| --- | --- |
| 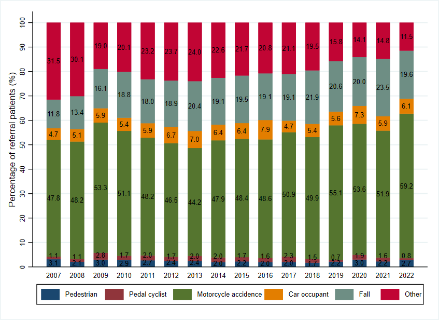  (c) | 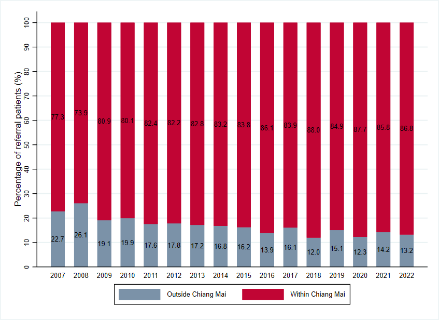  (d) |
| 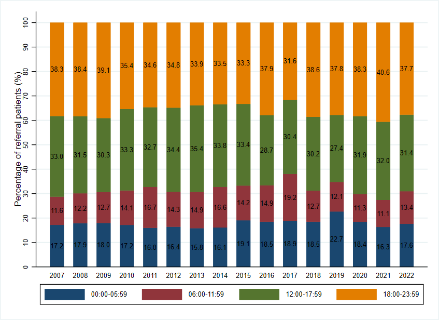  (e) | 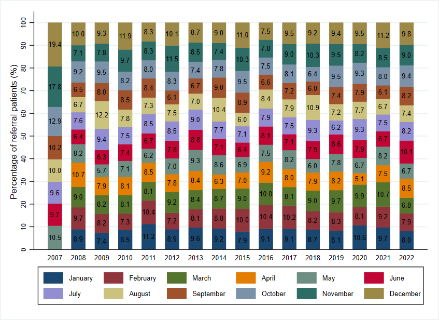  (f) |

**Supplementary Fig. S2** Percentages of study characteristics by year: (a) age group, (b) occupation, (c) mechanism, (d) referral source, (e) time of referral, and (f) month

| 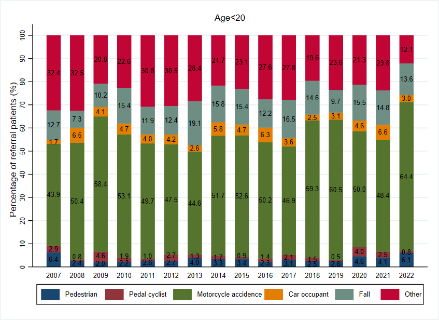  (a) | 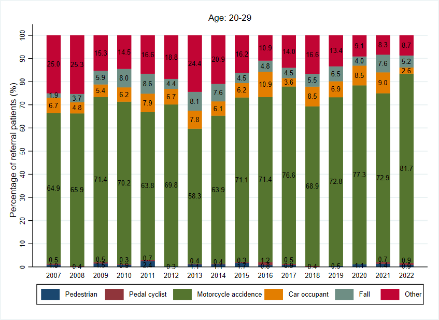  (b) |
| --- | --- |
| 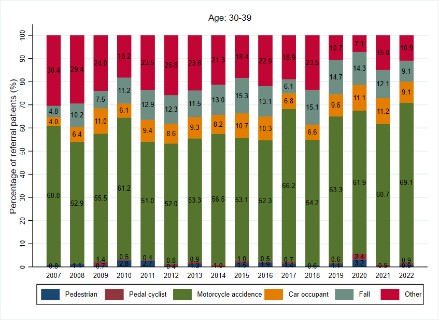  (c) | 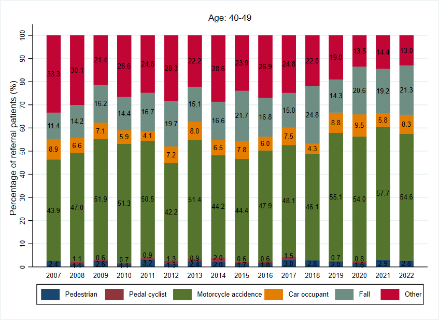  (d) |
| 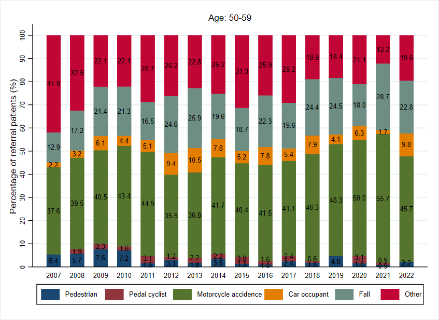  (e) | 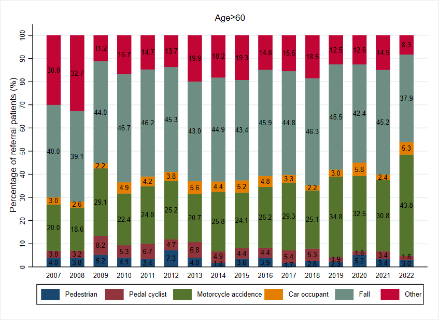  (f) |

**Supplementary Fig. S3** Population percentage of mechanism of injury by aged group and year from 2007-2022: (a) < 20 years, (b) 20-29 years, (c) 30-39 years, (d) 40-49 years, (e) 50-59 years, and (d) ≥ 60 years

| 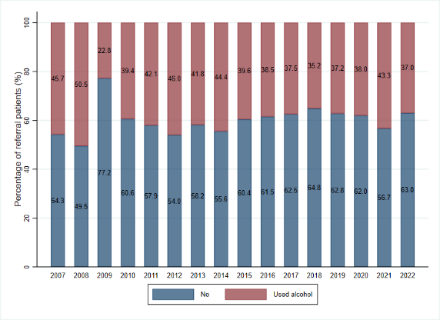  (a) | 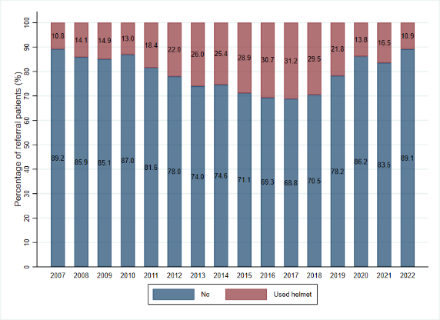  (b) |
| --- | --- |
| 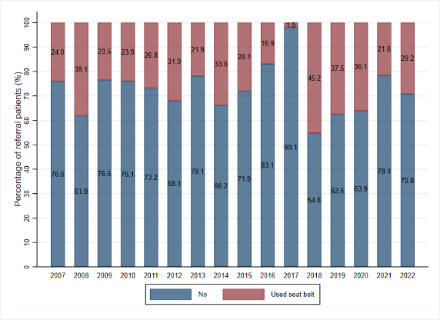  (c) | 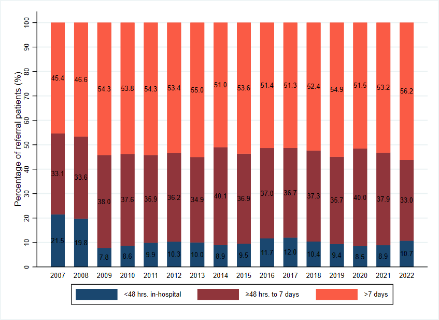  (d) |

**Supplementary Fig. S4** Percentages of study characteristics by time: (a) alcohol used, (b) not using a helmet, (c) not using a seat belt, (d) Time of in-hospital

| 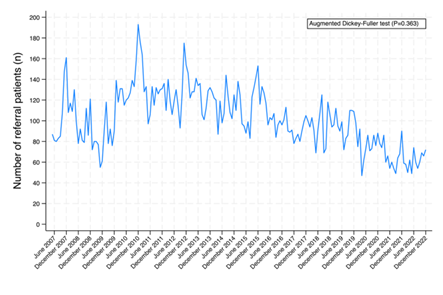  (a) | 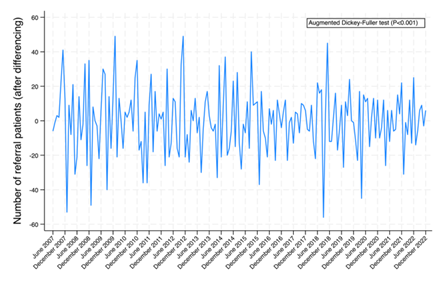  (b) |
| --- | --- |
| 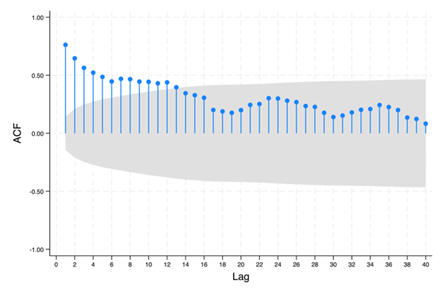  (c) | 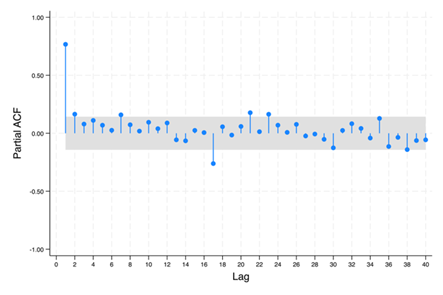  (d) |
| 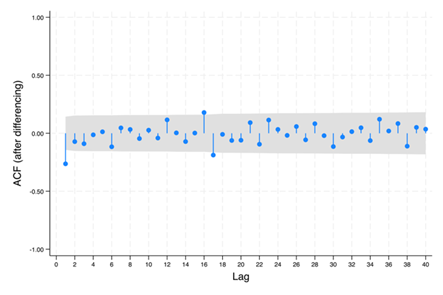  (e) | 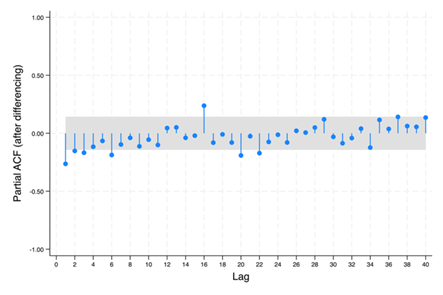  (f) |

**Supplementary Fig. S5** The original time series of the number of referred patients from May 2007 to December 2022, (a) the data is non-stationary according to the ADF test (*p-*value=0.36). (b) After performing the first difference transformation (d=1), the ADF test showed that the time series was stationary (*p-*value < 0.001). (c) ACF graph and (d) PACF graph of number of referral patients. (e) ACF graph and (f) PACF graph of time series after differencing transformation


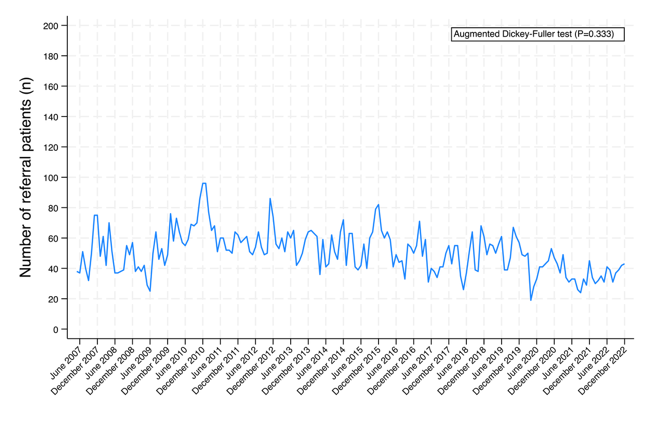

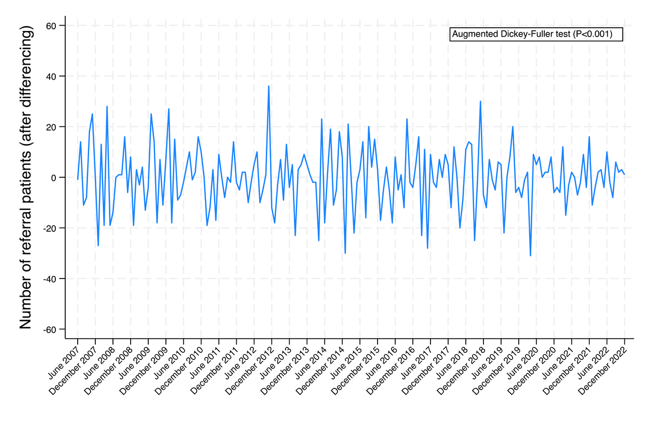


1. (b)


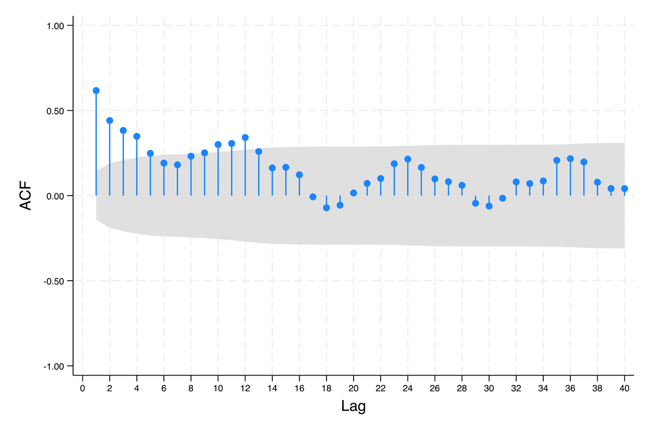

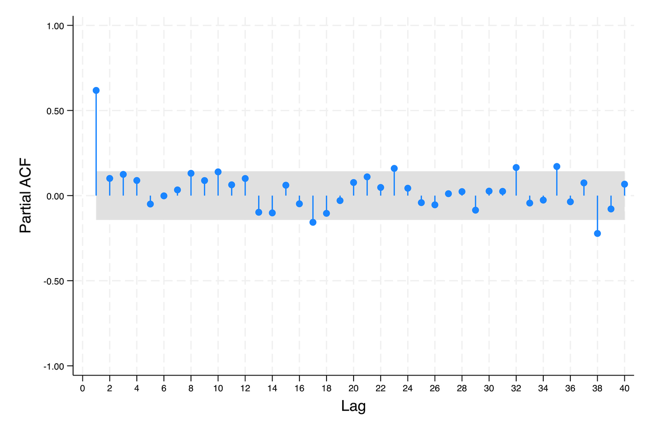


(c) (d)


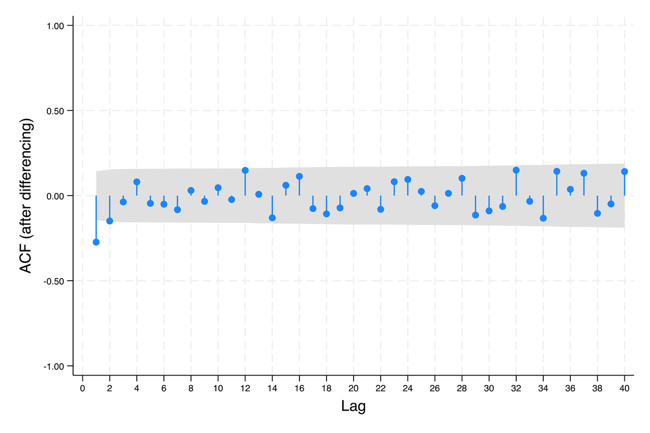

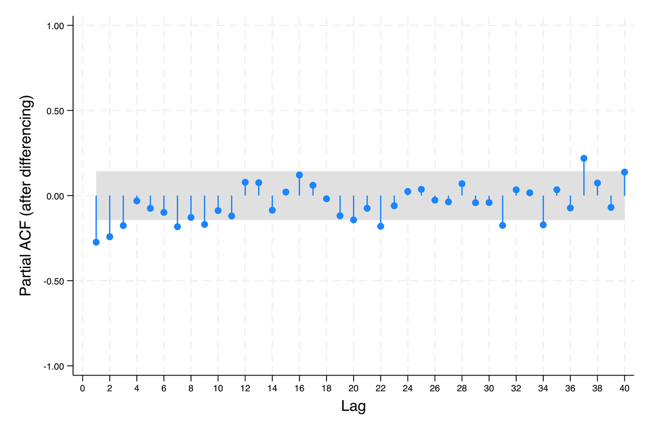


(e) (f)

**Supplementary Fig. S6** Motorcycle crash (a) the data is non-stationary (b) after performing the first difference transformation (d=1), (c) ACF graph and (d) PACF graph of motorcycle (e) ACF graph and (f) PACF graph of time series after differencing transformation


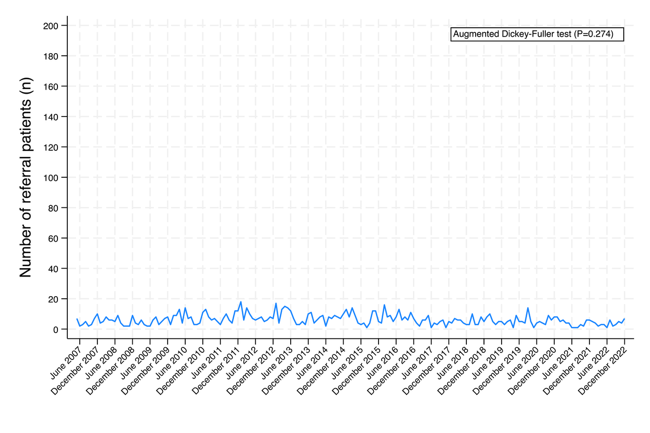

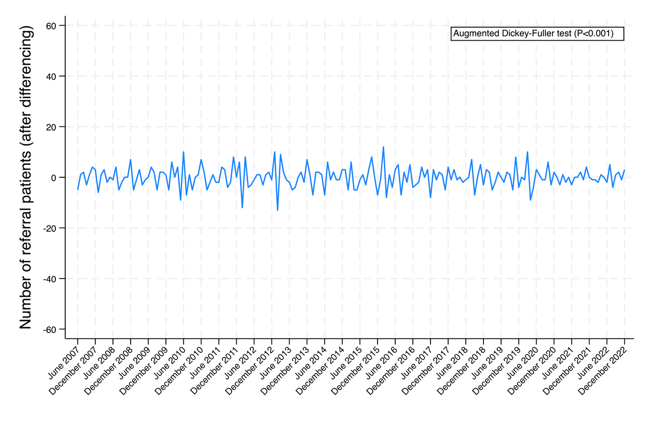


1. (b)


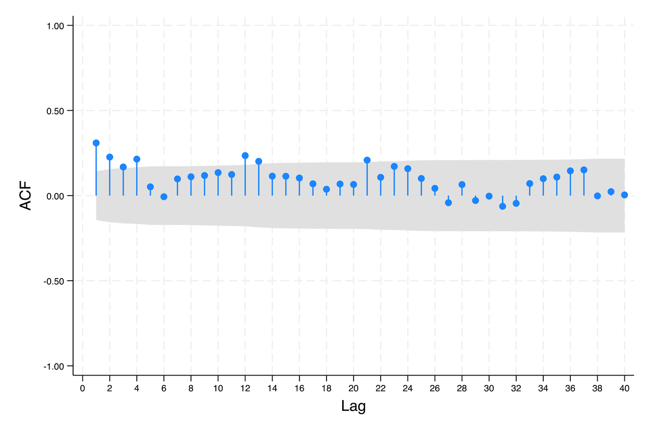

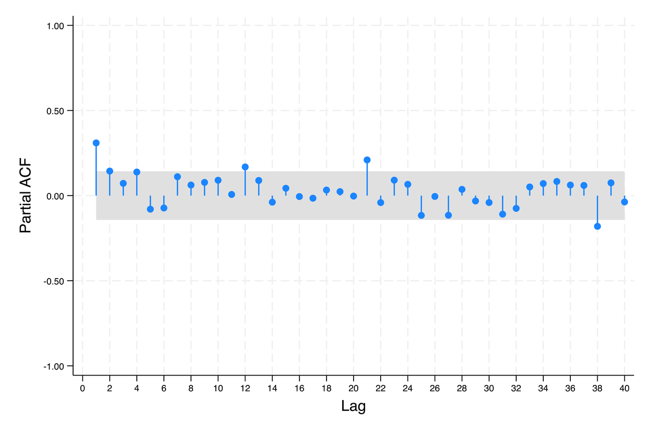


(c) (d)


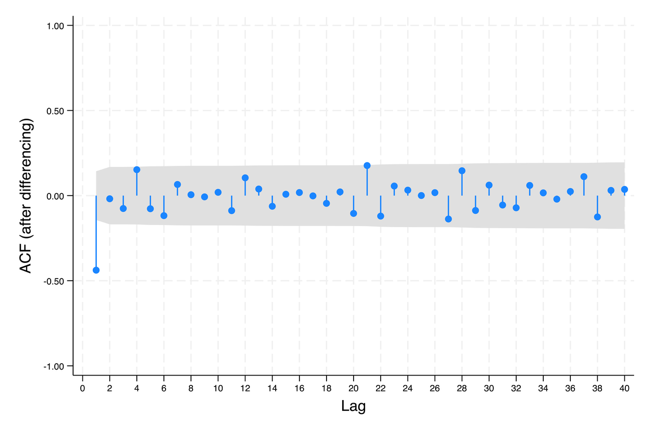

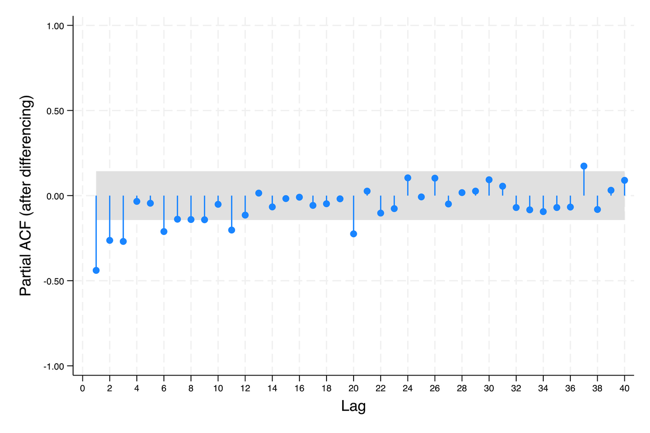


(e) (f)

**Supplementary Fig. S7** Car crash (a) the data is non-stationary (b) after performing the first difference transformation (d=1), (c) ACF graph and (d) PACF graph of Car. (e) ACF graph and (f) PACF graph of time series after differencing transformation


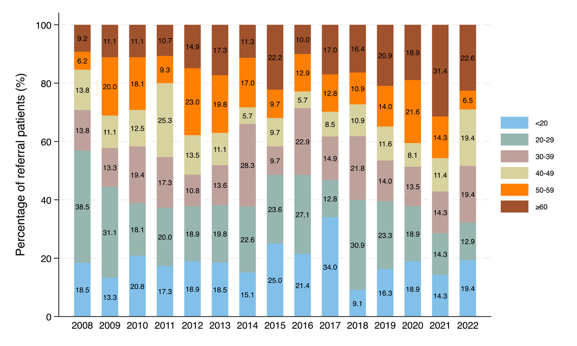

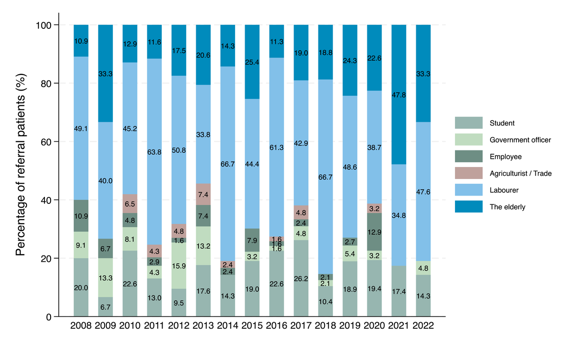


(a) (b)


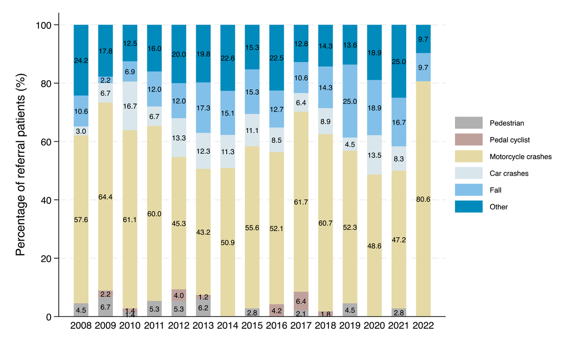

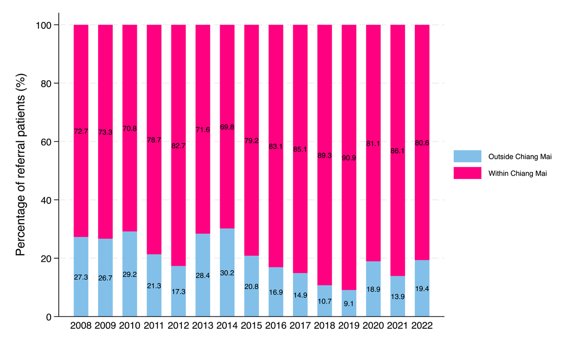


(c) (d)


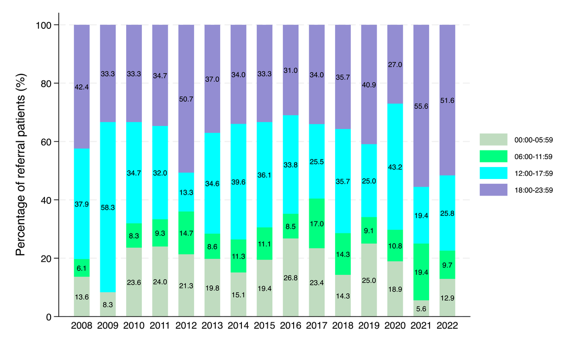


(e)

## **Supplementary Fig.** **S8** Percentages of study characteristics by year among referred patients during the “Seven Dangerous Days” of Songkran and New Year: (a) age group, (b) occupation, (c) mechanism of injury, (d) referral source, and (e) time of referral


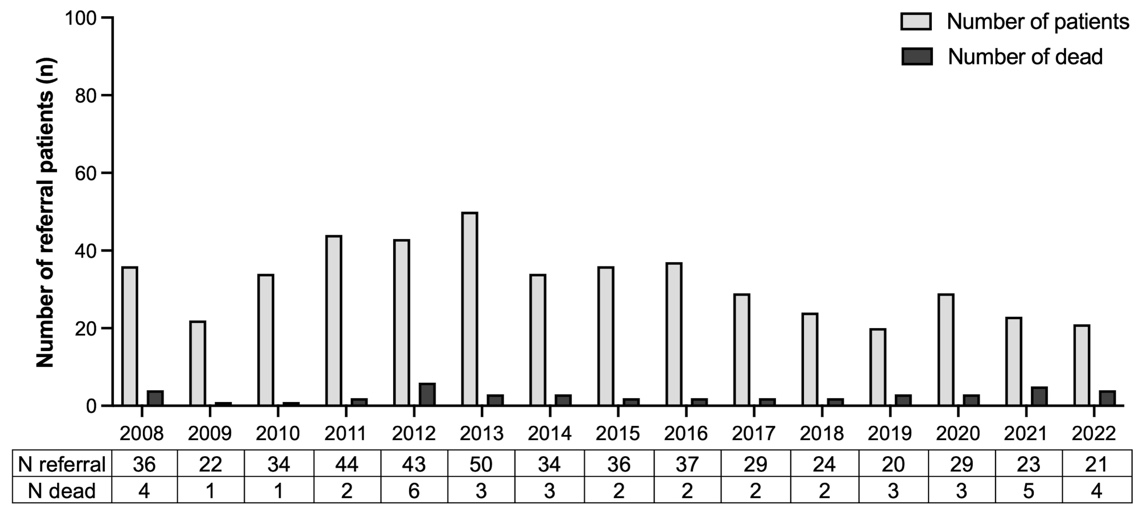


(a)


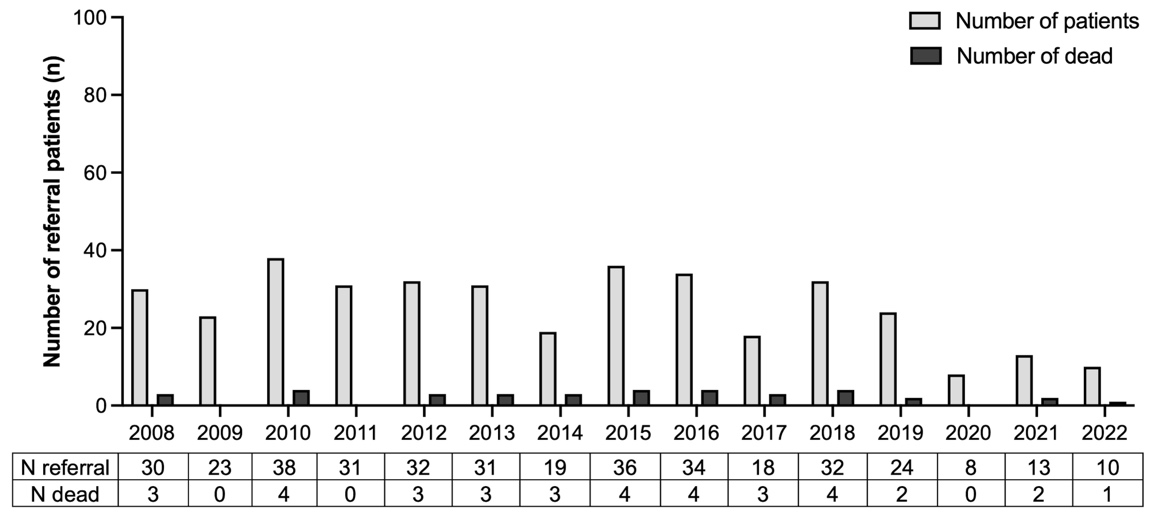


(b)

**Supplementary Fig.** **S9** Number of referred patients and deaths during the 7 Dangerous Days in Thailand: (a) New Year period, and (b) Songkran period


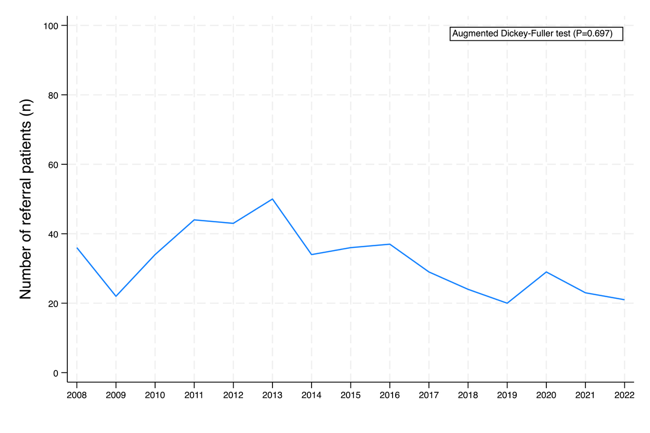

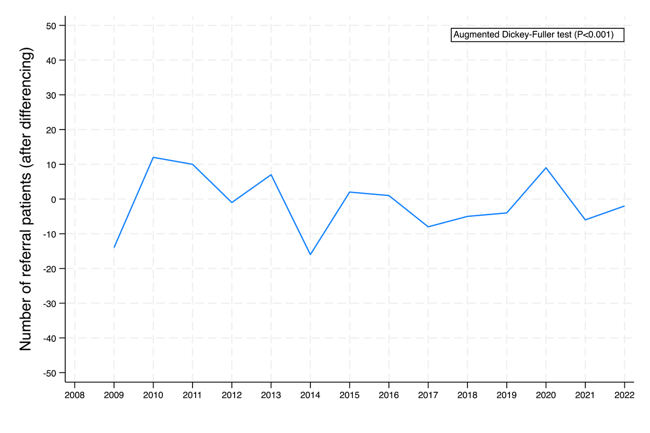


1. (b)


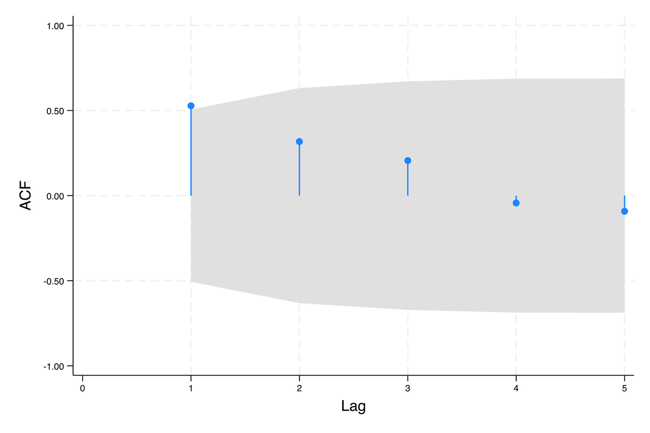

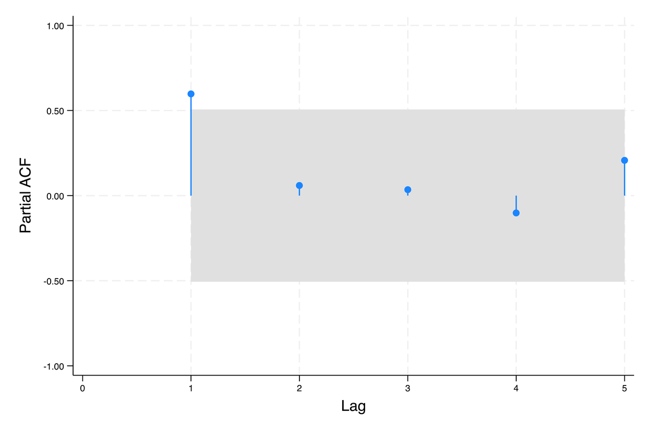


(c) (d)


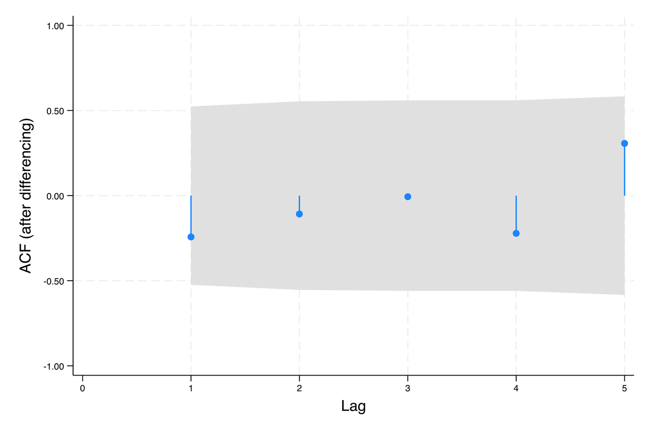

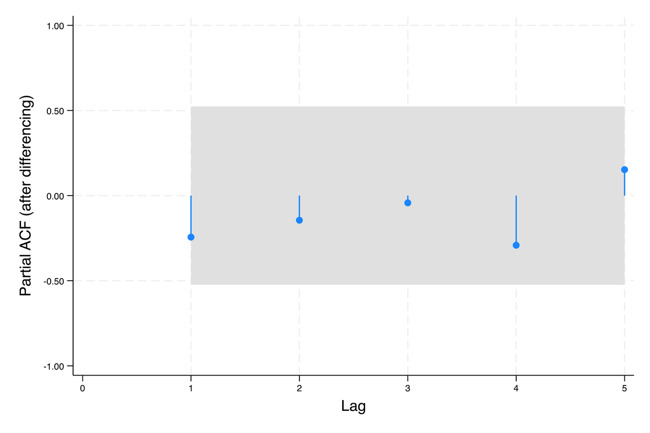


(e) (f)

**Supplementary Fig. S10** New Year (a) the data is non-stationary (b) After performing the first difference transformation (d=1), (c) ACF graph and (d) PACF graph of New Year (e) ACF graph and (f) PACF graph of time series after differencing transformation


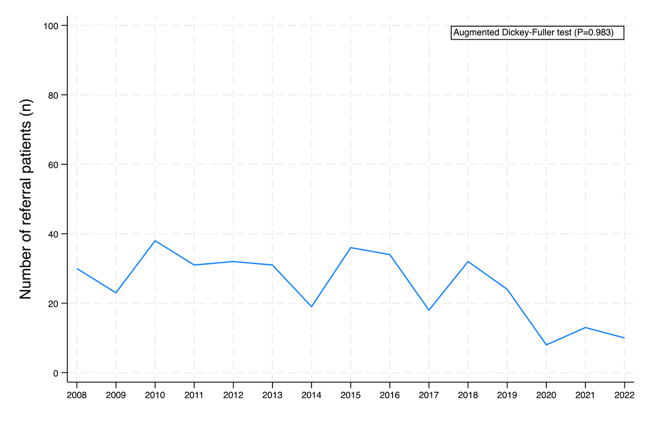

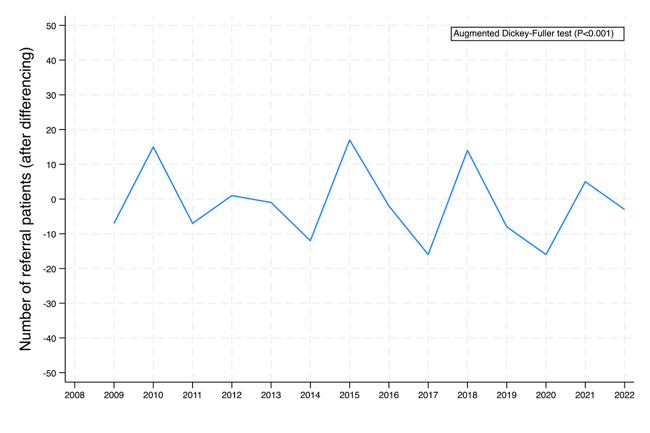


1. (b)


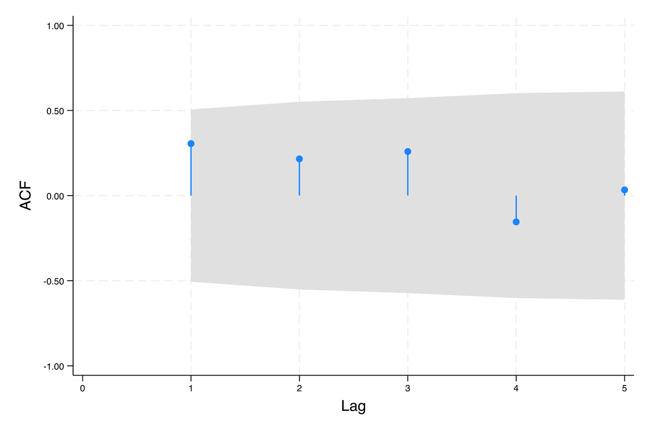

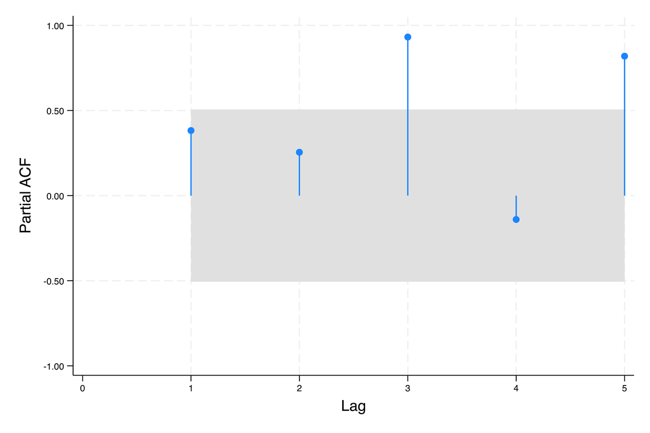


(c) (d)


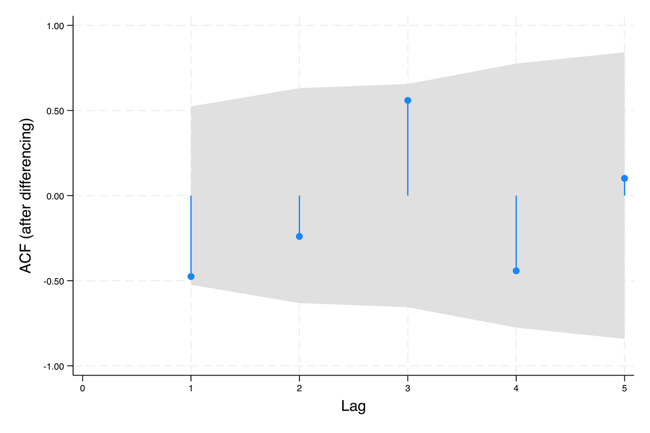

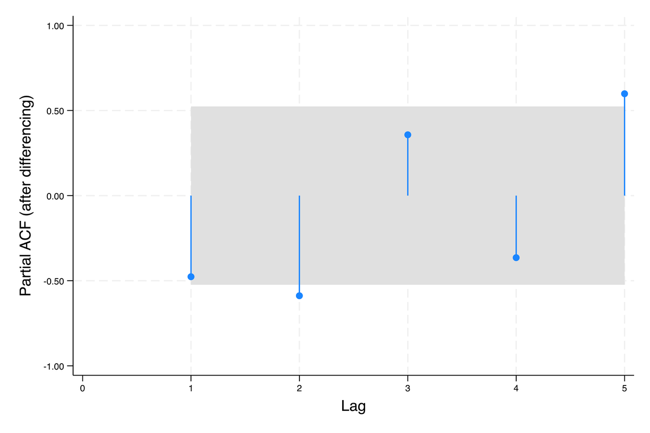


(e) (f)

**Supplementary Fig. S11** Songkran (a) the data is non-stationary (b) After performing the first difference transformation (d=1), (c) ACF graph and (d) PACF graph of Songkran (e) ACF graph and (f) PACF graph of time series after differencing transformation
